# Preclinical Evaluation of AZD6422, an Armored Chimeric Antigen Receptor T Cell Targeting CLDN18.2 in Gastric, Pancreatic, and Esophageal Cancers

**DOI:** 10.1158/1078-0432.CCR-24-1853

**Published:** 2024-09-25

**Authors:** Allison M. Barrett, Zachary T. Britton, Rosa A. Carrasco, Shannon Breen, Maria A.S. Broggi, Amanda L. Hatke, Benjamin Clark, Chunning Yang, Sandrina Phipps, Lorenzo Ortiz, Brianna Janocha, Peter Zanvit, Nicolas A. Giraldo, Philip L. Martin, Jean-Martin Lapointe, Nathalie Harder, Georgina H. Cornish, Bala N.N.R. Attili, Yariv Mazor, Melissa Damschroder, Mark Cobbold, Gordon Moody, Emily E. Bosco

**Affiliations:** 1 Early Oncology Research, Gaithersburg, Maryland.; 2 Biologics Engineering, Gaithersburg, Maryland.; 3 Oncology Translational Medicine, Gaithersburg, Maryland.; 4 Translational Pathology, Oncology R&D, AstraZeneca, Gaithersburg, Maryland.; 5 Clinical Pharmacology and Safety Sciences, BioPharmaceuticals R&D, AstraZeneca, Cambridge, United Kingdom.; 6 Computational Pathology, AstraZeneca, Munich, Germany.

## Abstract

**Purpose:**

Claudin 18.2 (CLDN18.2) is a surface membrane protein that is crucial for maintaining tight junctions in gastric mucosal cells and is highly expressed in gastric, esophageal, and pancreatic cancers. Thus, CLDN18.2 is suited for exploration as a clinical target for chimeric antigen receptor T-cell (CAR-T) therapy in these indications. Although CAR-T therapies show promise, a challenge faced in their development for solid tumors is the immunosuppressive tumor microenvironment, which is often characterized by the presence of immune and stromal cells secreting high levels of TGFβ. The addition of TGFβ armoring can potentially expand CAR-T activity in solid tumors. We report on the preclinical development of a CLDN18.2-targeting CAR-T therapy showing effectiveness in patient models with CLDN18.2-positive gastric, esophageal, and pancreatic tumors.

**Experimental Design:**

The lead lentivirus product contains a unique single-chain variable fragment; CD28 and CD3z costimulatory and signaling domains; and dominant-negative TGF-β receptor armoring, enhancing targeting and safety and counteracting suppression. We developed a shortened cell manufacturing process to enhance the potency of the final product AZD6422.

**Results:**

AZD6422 exhibited significant antitumor activity and tolerability in multiple patient-derived tumor xenograft models with various CLDN18.2 and TGF-β levels, as determined by IHC. The efficacy of armored CAR-T cells in tumor models with elevated TGFβ was increased *in vitro* and *in vivo*. *In vitro* restimulation assays established greater persistence and cytolytic function of AZD6422 compared with a traditionally manufactured CAR-T.

**Conclusions:**

AZD6422 was safe and efficacious in patient-derived, CLDN18.2-positive murine models of gastrointestinal cancers. Our data support further clinical development of AZD6422 for patients with these cancers.

**Translational Relevance**

AZD6422 is a novel armored chimeric antigen T cell generated with an optimized manufacturing process to target CLDN18.2 antigen–expressing gastrointestinal tumors and is currently under evaluation in a phase I investigator-initiated trial (ClinicalTrials.gov Identifier: NCT05981235). We report on the preclinical development and activity of AZD6422 in patient-representative tumor models spanning a range of CLDN18.2 antigen expression, including models of post-chemotherapy relapse. *In vitro* assays demonstrated prolonged persistence in serial restimulation assays with the optimized manufacture protocol, and *in vivo* model systems demonstrated robust antitumor activity and tolerability after a single infusion of AZD6422. The unique single-chain variable fragment, the addition of armoring, and an optimized manufacturing protocol poise AZD6422 to be successful in the clinic for cancer indications with high unmet needs.

## Introduction

Gastrointestinal (GI) tract cancers, including those of the stomach, esophagus, and pancreas, account for approximately one quarter of cancer incidence and close to one-third of cancer-related mortality worldwide (1, 2). Many factors account for this high mortality, including diagnosis at an advanced stage, intra- and intertumor heterogeneity (3), and the immunosuppressive tumor microenvironments (TME) that often accompany these indications (4, 5). The past 50 years have seen a continuous decline in incidence due to improved diet and decreased smoking rates across the globe in general, as well as more effective treatment of *Helicobacter pylori* infection in Asian countries in particular (6). Nevertheless, gastric and gastroesophageal junction cancers remain a global public health problem. Likewise, pancreatic cancer remains an area of high unmet need; it is expected that pancreatic cancer will become the second leading cause of cancer-related deaths in the United States by 2030, with a 5-year survival rate of merely 7.2%. As a result, many therapies, including chimeric antigen receptor T (CAR-T) cells, are in development for these indications.

CAR-T therapy is a cell-based approach that uses engineered T cells to eliminate antigen-presenting target cells independently of the MHC. Second-generation CAR-T cells are the most clinically used design to date. These CAR-T cells are engineered to incorporate the intracellular signaling domain of a T-cell costimulatory receptor, commonly derived from either CD28 or 4-1BB, to enhance proliferation and cytokine secretion upon antigen recognition. CAR-T cells have exhibited impressive and clinically transformative outcomes in B-cell malignancies (7, 8), but hematologic malignancies account for a small fraction of the overall total incidence and mortality caused by cancer (9). Translation to the solid tumor setting has been a challenge (10), and augmentations to boost the efficacy of CAR-T cells in solid tumors are required.

The foundation of a successful CAR-T therapy is the selection of the target tumor-associated antigen, which ideally displays high, homogeneous expression on tumor cells and limited expression in normal tissue. However, finding antigens with this ideal profile has proved to be challenging, as most solid tumor targets are also found in normal tissues (11). Claudin 18 (CLDN18) is a well-characterized four-transmembrane protein and one of a family of at least 27 CLDNs that regulate cell–cell adhesion, cell permeability, and cell polarity in epithelial cells in a tissue-restricted manner (12). The gastric-specific isoform CLDN18.2 has normal tissue expression that is mostly restricted to differentiated cells of the gastric mucosa. It is also highly prevalent in GI cancers (13, 14) and, importantly, in both primary and metastatic lesions (15), which makes it a promising clinical target. Indeed, CLDN18.2 is currently being evaluated clinically via multiple therapeutic modalities (16–18).

Another obstacle for solid tumor therapies is the highly immunosuppressive TME of these cancers. In GI tumor subtypes, the TME contains suppressive immune cells, such as regulatory T cells, myeloid-derived suppressor cells, and tumor-associated macrophages, which promote tumor cell proliferation, metastasis, and secretion of inhibitory cytokines. The latter include IL4, IL10, and TGFβ, which can hinder antitumor T-cell function (19, 20). Other solid tumors, such as pancreatic ductal adenocarcinoma (PDAC), have additional suppressive features, such as a dense stromal barrier and harsh metabolic conditions resulting from elevated hypoxia and nutrient scarcity (11).

Unlike other therapeutic modalities, cell-based therapies can be engineered beyond the incorporation of the CAR construct to mitigate the TME. Examples are so-called “armoring” approaches which enable CAR-T cells to evade or alter the TME or to overcome tumor antigen escape through the secretion of cytokines, expression of cytokine receptors on the CAR-T cellular surface, or manipulation of T-cell signaling through switch receptors (21, 22). Another extensively explored armoring strategy is the inclusion of a dominant-negative TGFβ receptor II (dnTGFβRII). This engineered receptor maintains TGFβ binding and dimerization but abolishes downstream inhibitory signaling, which enhances CAR-T proliferative capacity and cytokine secretion even in the presence of inhibitory levels of TGFβ. Many CAR-T cells using this armoring approach in various cancer indications have been examined to date (23–26).

In the setting of B-cell malignancies, CAR-T expansion and persistence correlate with a durable response (8); therefore, an additional means to achieve effective responses in solid tumors may lie in generating CAR-T cells with the capacity to expand and persist to prevent relapse (27). The phase I analysis after administration of the CLDN18.2 CAR-T product CT041 showed promising antitumor efficacy and, importantly, a tolerable safety profile in patients with gastric cancer (18). However, the duration of response was short [median progression-free survival (PFS) of 4.4 months] and the median persistence after the first infusion was 28 days, which may indicate room for improvement of cell therapeutics targeting CLDN18.2 (18). Extensive analysis of clinical response in the setting of B-cell malignancies has focused on the impact of the costimulatory domain used in the CAR-T product, and many reports have indicated that CAR-T cells containing a 4-1BB costimulatory domain have longer persistence than those with CD28 (28). However, this finding may need to be more well studied in regard to the potential impact of persistence and safety in the solid tumor setting, as some research, though limited in scope, may indicate that the use of a short-lived effector T cell may be favored in murine models of solid tumors (29, 30). It has also been established that the selection or generation of CAR-T cells with a less differentiated stem cell memory phenotype can endow the CAR-T product with greater self-renewal and proliferative capacity, which may lead to longer persistence and a stronger antitumor response (31, 32). This favorable CAR-T phenotype can be generated through several methods: (i) starting with a selected T-cell population; (ii) incorporating metabolites, antibodies, or small molecules during *ex vivo* expansion and manufacturing to preserve T stemness; and/or (iii) employing a shortened manufacturing process (27, 32–35). Traditionally, isolated T cells undergo multiple doublings during the expansion period until sufficient CAR-T cells are achieved to formulate a dose. Optimization of the manufacturing strategy to produce an infusion product enriched in these less differentiated cells is a means to generating a more persistent and metabolically enhanced CAR-T with the potential for augmented antitumor activity (27, 36).

AZD6422 is an autologous CLDN18.2-targeting CAR-T that is currently under clinical development (ClinicalTrials.gov identifier: NCT05981235) and incorporates defensive dnTGFβRII armoring and an optimized, shortened manufacturing process. Here, we report the development of a CAR T-cell product with a less differentiated and clinically favorable phenotype and the potential to shorten the time from apheresis to infusion in the clinical setting. We also report on the preclinical efficacy and tolerability of AZD6422 in multiple clinically relevant patient-derived xenograft (PDX) tumor models, the results of which support further clinical development of this armored CAR-T product.

## Materials and Methods

### Cell lines and cell line generation

All cells were cultured according to manufacturers’ recommendations and were maintained at 37°C in a humidified atmosphere at 5% CO_2_. Cell lines were authenticated by the AstraZeneca cell bank with short tandem repeat analysis using CellCheck and tested by IMPACT PCR Profile for *Mycoplasma* and other contaminants on receipt of cell lines and banking (IDEXX BioAnalytics). Cells from the bank were used for 10 passages post-thaw. ASPC1 (RRID: CVCL_0152), BxPC3 (RRID: CVCL_0186), and HEK293 cell lines were obtained from the ATCC. NUGC4 (RRID: CVCL_3082) was obtained from Riken BioResource Research Center. SNU601 (RRID: CVCL_0101) was obtained from the Korean Cell Line Bank. The PaTu8988s cell line (“unsorted,” RRID: CVCL_1846) and DAN-G (RRID: CVCL_0243) were obtained from the DSMZ collection. IM95 (RRID: CVCL_2961) was obtained from the Japanese Collection of Research Bioresources Cell Bank.

To generate CLDN18 variant cell lines, DNA encoding human CLDN18.2 (UniProt: P56856-2), human CLDN18.1 (UniProt: P56856-1), human CLDN18.2 M149L, human CLDN18.2 Q29M, human CLDN18.2 N37D, human CLDN18.2 A42S, human CLDN18.2 N45Q, human CLDN18.2 Q47E, human CLDN18.2 E56Q, human CLDN18.2 G65P, human CLDN18.2 L69I, mouse CLDN18.2 (UniProt: P56857-3), and mouse CLDN18.1 (UniProt: P56857) were obtained from Integrated DNA Technologies and transferred into the pCDH-CMV-MCS-EF1α-Puro Cloning and Expression Lentivector system (System Biosciences). The puromycin resistance gene was used for selection. DNA encoding human and mouse CLDN18.2 was transferred into a modified lentivirus pCDH1-CMV-MCS-EF1α-Puro-T2aA-GFP vector. Cells were selected with 2 μg/mL puromycin, expanded, and banked. ASPC1, HEK293, BxPC3, and NUGC4 cells were similarly transduced with lentivirus and selected with puromycin, although the medium and culture requirements were different. All cell lines engineered to express CLDN18.2 or CLDN18.1 are indicated herein as “+CLDN18.2” or “+CLDN18.1.”

The PaTu8988s high sort (HS) cell line was bulk-sorted using a BD FACSAria system (BD Biosciences). PaTu8988s CLDN18.2 knockout (KO) cells were generated by using a combination of three pooled CLDN18 guide RNA (Gene Knockout Kit, version 2, Synthego) and Cas9 (Integrated DNA Technologies). Ribonucleoprotein complexes were assembled according to the manufacturer’s suggested protocol (9:1 ratio of single-guide RNA to Cas9). Cells and precomplexed ribonucleoproteins were electroporated in an RUO OC-25x3 cassette (MaxCyte) using an ExPERT GTx electroporation instrument (MaxCyte). PaTu8988 KO cells underwent three rounds of this protocol.

### Lentivirus preparation

Lentivirus vectors were co-transfected with pPACKH1 (catalog no. LV500A-1; System Biosciences) into a suspension of HEK293 cells and incubated for 48 hours at 37°C with 8% CO_2_ at 125 rpm. On day 2 after transfection, the suspension of HEK293 cultures was transferred to a 50-mL conical tube and centrifuged for 5 minutes at 2,500 rpm. Culture supernatants containing lentivirus were filtered, and then PEG-it virus precipitation solution (catalog no. LV825A-1; System Biosciences) was added according to the manufacturer’s protocol and incubated overnight at 4°C. The supernatant was centrifuged at 1,500 × *g* for 30 minutes at 4°C. Pelleted lentivirus was resuspended in 600 μL of Opti-MEM (catalog no. 31985062; Thermo Fisher Scientific), aliquoted in cryovials, and stored at −80°C. In some instances, lentivirus was produced by Lentigen Technology, using proprietary vectors and methods with the sequences provided.

### Cell-based phage selections for isolation of CLDN18.2-specific leads

Engineered HEK293 cells plus human CLDN18.2 were used for the selection of CLDN18.2-reactive phage from the recombinant framework scFv phage library, a naïve, synthetic, VH-VL single-chain variable fragments (scFv) library that is based on the IGHV1-69*01 and IGLV1-44*01 germlines. CDR H1-2 and CDR L1-2 contain wholly germline sequences, and the library diversity (1e9) results from nine randomized amino acids in CDR H3 (ARXXXXXXXXDX) and five randomized amino acids in CDR L3 (AAWDXXXXXVV).

Three rounds of phage selections were performed, exhibiting round-to-round enrichment of CLDN18.2-reactive leads. Round 2 selection outputs exhibited favorable specificity and diversity profiles and were used as the basis for large-scale screening.

### Flow cytometry assessments

For flow cytometry (FC) assessments listed below, indicated cell lines at 80% to 90% confluency were harvested with TrypLE Express Enzyme (Thermo Fisher Scientific) and stained at 4°C with indicated anti-CLDN18.2 detection reagent in FACS buffer (1× PBS plus 2% FBS) for 30 minutes, and if not fluorophore-conjugated, stained with Alexa Fluor 647 AffiniPure F(ab′)_2_ fragment goat anti–human IgG (Jackson ImmunoResearch) for 30 minutes after washing. After the indicated staining protocols were carried out, cells were washed and then resuspended in FACS buffer supplemented with 4′,6-diamidino-2-phenylindole. Antibody binding was assessed using either a FACSymphony (BD Biosciences) or a MACSQuant (Miltenyi Biotec) flow cytometer. Data were analyzed using FlowJo software (Tree Star). All assays were run in a round-bottomed, 96-well plate, and centrifuge steps were conducted at 1,200 rpm.

#### Specificity by FC

Candidate scFvs from phage selections were converted to scFv-Fc format, and CLDN18.2 isoform reactivity and specificity were assessed using HEK293 + CLDN18.2 and HEK293 + CLDN18.1 (human and mouse) and PaTu8988s HS.

#### Affinity by FC

The triaged CLDN18.2-specific scFvs were converted to IgG1 format and then characterized for affinity and cross-reactivity using HEK293 + CLDN18.2 (human or mouse) with titrated antibody (0–533 nmol/L). Histograms of median fluorescence intensity (MFI) were used to calculate the geometric mean for each antibody at a given concentration (FlowJo). Geometric means were plotted in Prism (GraphPad Software) and used to calculate the binding EC_50_.

#### Epitope assessment by FC

HEK293 + huCLDN18.2 were labeled with CellTrace CFSE Cell Proliferation kit (catalog no. C34554; Thermo Fisher Scientific) and then mixed 1:1 with unlabeled HEK293 with variant CLDN18.2 in a 96-well, round-bottomed plate. For each antibody characterized, eight wells were required to determine CLDN18.2 specificity with gates for live/dead cells; FITC-positive [CLDN18.2 wild-type (WT)] and FITC-negative (CLDN18.2 variant) cells and 10 to 20 μg/mL antibody was used. For variants that do not contribute to the binding epitope, the FITC-positive and -negative cell populations exhibited equivalent antigen-presenting cell MFI. For variants that influence or abolish binding epitope, the FITC-positive cell population exhibited a stronger antigen-presenting cell signal.

#### M149L binding assessment by FC

HEK293 + CLDN18.2_M149L were stained with 10 μg/mL CLDN18.2 antibodies side by side with HEK293 + CLDN18.2 WT, and MFI histograms were used to assess maintained or loss of binding.

#### Quantitative cell surface receptor density by FC

An alternate (noncandidate clone) anti–CLDN18.2 IgG conjugated to Alexa Fluor 647 was used at 0.5 μg/mL, and all cells were run at the same time and in triplicate. For quantitation purposes, Quantum Simply Cellular beads (Bangs Laboratories) were included in each assay and stained in the same manner as cancer cells. After data acquisition, MFI was translated to antibody-binding capacity values using the QuickCal analysis template (Bangs Laboratories).

### CAR conversion

To generate lentivirus expression vectors encoding CLDN18.2-reactive CARs, DNA encoding clone 2, clone 5, and clone 9 scFv sequences were amplified by PCR from pSpliceV4 and gel purified. scFv encoding PCR products were then assembled into appropriately digested pESRC-CD33 leader-MCS-IgG4P-CD28 TM-4-1BB-CD3z-T2a-GFP or, for clone 9, pESRC-CD33 leader-MCS-IgG4P-CD28 TM-CD28-CD3z-T2a-mCherry. These constructs contain sequences that encode the CD33 leader sequence, the IgG4P hinge with the S228P mutation (IgG4P), the transmembrane domain of CD28, either the 4-1BB or the CD28 cytosolic domain, the CD3z cytosolic domain, the self-cleaving T2a peptide, and GFP, mCherry, or dnTGFβRII.

### CAR-T manufacture and phenotyping

Traditional manufacture of CAR-T cells used the protocol as previously reported (25). Cells were cultured at 37°C in a humidified incubator with 5% CO_2_ in an AIM-V medium containing 5% human AB serum (Valley Biomedical) and human IL2 (300 IU/mL; PeproTech). Lentivirus was added at a multiplicity of infection of five with the addition of polybrene (1 μg/mL). CD3/CD28 Dynabeads (Thermo Fisher Scientific) were magnetically removed at 72 hours, and cells were split as necessary during the expansion period to maintain a cell density of 0.5e6 to 1.0e6 per mL. Cells were generally used immediately between 9 and 12 days after transduction or cryopreserved for extended storage in CryoStor CS10 Freeze Media (STEMCELL Technologies).

STAR-T manufacture of CAR-T cells used the protocol as previously reported (25). Due to the shortened culture and proliferation time, the starting number of donor T cells was increased for STAR-T manufacture. STAR-T complete medium consists of X-Vivo 15 medium (Lonza) plus IL2 (Miltenyi Biotec), IL21 (Miltenyi Biotec), and 1× ITSE + A - Blood-free culture medium supplement (InVitria). For STAR-T manufacture, healthy donor total T cells in complete medium were placed in a polycarbonate Erlenmeyer flask with a vent cap (Corning) and activated by the addition of Transact (1:17.5, vol/vol; Miltenyi Biotec) at 37°C and 5% CO_2_ with agitation. The next day, lentivirus was added at multiplicity of infection of 1.5. Cells were monitored for viability and medium was exchanged each day to maintain cells at 1.5e6 per mL during expansion. Cells were generally used immediately on day 4 after transduction or were cryopreserved for extended storage as noted above.

Transduction efficiency was determined by FC with an anti–Fab AF647 (catalog no. 109-606-066; Jackson ImmunoResearch), an anti–clone 9 scFv paratope antibody conjugated to Alexa Fluor 647 (generated by GenScript PROBIO, “clone 9 scFv paratope”), or GFP/mCherry co-expressed with CAR gene in the lentivirus vector. To determine the surface expression of dnTGFβRII, an anti-TGFβRII PE antibody (catalog no. 399703; BioLegend) was used. Cells were washed three times in FACS buffer and stained with the abovementioned reagents for 30 minutes at 4°C in the dark. Cells were then washed an additional three times and resuspended in FACS buffer containing 4′,6-diamidino-2-phenylindole to gate on live/dead cells. To phenotype CAR-T cells by FC, panels comprising some variation of the antibodies listed here were used: anti–human CD45 (catalog no. 304014, HI30), CD3 (catalog no. 300434, UCHT1), CD8 (catalog no. 301028, RPA-T8), CD45RO (catalog no. 304238, UCHL1), and CCR7 (catalog no. 353204, G043H7; all from BioLegend); CD8 (catalog no. 555366, RPA-T8), CD4 (catalog no. 566923, SK3), CD4 (catalog no. 560650, RPA-T4), and CD62L (catalog no. 565219, SK11; all from BD Biosciences); and anti–clone 9 scFv paratope and anti-TGFβRII. Dead cells were excluded from analysis using Live/Dead Fixable Blue Dead Cell Stain Kit (Thermo Fisher Scientific, L23105) by staining cells in PBS for 15 minutes at room temperature according to the manufacturer’s protocol. For all FC assays, acquisition was performed with a FACSymphony instrument (BD Biosciences), and data were analyzed using FlowJo software.

### Western blotting

A pure CAR-T cell population was sorted via FACS. After 24 hours of serum starvation, purified CAR-T cells were co-cultured with 1 ng/mL recombinant human TGFβ for various time periods and then lysed on ice in RIPA +1× protease and phosphatase inhibitors for protein detection. Lysates were run through SDS-PAGE according to the manufacturer’s protocol with a Novex NuPAGE gel (Thermo Fisher Scientific) and then transferred to a nitrocellulose membrane using an iBlot Dry Blotting System (Thermo Fisher Scientific). p-SMAD2/3 (catalog no. 8828S; Cell Signaling Technology), total SMAD2/3 (catalog no. 8685S; Cell Signaling Technology), and β-actin (catalog no. A3854; Sigma Aldrich) as a loading control were detected via horseradish peroxidase–conjugated antibodies and ultrasensitive enhanced chemiluminescent substrate (Thermo Fisher Scientific) using the ImageQuant biomolecular imaging system (Cytiva Life Sciences).

### CAR-T metabolism

CAR-T cells were thawed and resuspended in X-Vivo 15 medium for 1 hour. Cells were stained with 1 μg/mL anti–clone 9 scFv paratope reagent at room temperature for 10 minutes, and then, CAR+ cells were isolated using anti–Alexa Fluor 647 Microbeads (Miltenyi Biotec) according to the manufacturer’s instructions. For Seahorse Xfe96 Analyzer (Agilent Technologies) analysis, cells were plated onto 96-well Seahorse XFe96 poly-D-lysine cell culture microplates at 2e5 per well (catalog no. 103799-100; Agilent Technologies). The glycolysis stress test (catalog no. 1030020-100; Agilent Technologies) was performed by measuring the extracellular acidification rate (mpH/minute) at steady state and after sequential injection of D-glucose (10 mmol/L; 1 µmol/L), and 2-deoxy-D-glucose (50 mmol/L; all from Agilent Technologies). The mitochondrial stress test (catalog no. 103015-100; Agilent Technologies) was performed by measuring the oxygen consumption rate (pmol/minute) at steady state and after sequential injection of 1.5-μmol/L oligomycin, 2-μmol/L carbonyl cyanide p-trifluoro-methoxyphenylhydrazone, rotenone, and antimycin A (0.5 μmol/L; all from Agilent Technologies). Experiments were run on the Seahorse XFe96 instrument with the following assay conditions: three cycles, 3 minutes mixture, and 3 minutes measurement.

### IHC

Human tissue samples were obtained as formalin-fixed, paraffin-embedded samples from various commercial suppliers (TriStar Technology; Analytical Biological Services; US Biomax), in full compliance with UK and US regulations and fully consented for research use by the donors with informed written consent. Collected mouse tissues were fixed in neutral-buffered formalin and processed to paraffin using routine methods. Prior to IHC staining, 4-μm-thick sections were taken from blocks and baked at 60°C for 1 hour. Staining for CLDN18.2 was performed using an automated Bond RX platform (Leica). After deparaffinization, antigen retrieval was performed in Bond ER2 Solution (Leica) for 30 minutes at 98°C, a peroxidase block was applied for 5 minutes, and then anti-CLDN18.2 antibody (clone EPR19202; Abcam) was incubated at room temperature for 60 minutes at 2.0 μg/mL. Detection was performed using a diaminobenzidine (DAB) chromogen kit (Polink-2 Plus, OriGene Technologies) with a hematoxylin counterstain. For TGFβ staining, antigen retrieval was done in Bond ER2 Solution for 20 minutes at 100°C, followed by peroxidase block for 10 minutes and S-Block 1/1 (DISC S Block RUO, catalog no. 05268931001; Roche Diagnostics) for 15 minutes. Anti–TGFβ1 antibody (clone EPR21143; Abcam) was incubated for 60 minutes at a concentration of 1.74 μg/mL (1:300), and detection was performed with DAB using the Bond Polymer Refine kit (Leica) with a hematoxylin counterstain.

Stained slides were digitally scanned using an Aperio Scanscope AT2 pathology slide scanner (Leica). Whole-slide images were reviewed by a pathologist assessing the cell types expressing CLDN18.2 and the intensity and cellular localization of staining. For tumor samples, CLDN18.2 expression was evaluated semiquantitatively using H-scores, which are calculated by multiplying the percentage of tumor cells staining positive by intensity on a scale of 1 to 3 (H-score = [%TC(+) 1+ × 1] + [%TC(+) 2+ × 2] + [%TC(+) 3+ × 3]). TGFβ IHC scores for PDX models were generated for tumor and stromal compartments. For tumors, the proportion of cells with any level of expression (0–4 scale) was multiplied by the intensity of staining (1–3 scale) to end at a 0 to 12 score. For the stromal component, the total amount of stromal cell presence varied among the tumor samples; the total presence of stromal cells (0–3 scale) was multiplied by the overall intensity of staining (1–3 scale) to obtain the stromal staining value (0–9). The tumor (0–12) and stromal (0–9) scores were added to obtain a total TGFβ score of 0 to 21, and models were separated into low, intermediate, and high based on calculations of the geometric mean. For patient tumor microarray (TMA) cores, TGFβ expression was measured by automatic image analysis, quantitative continuous scoring (37) of tumor cells and stromal cells was analyzed separately for each TMA core and reviewed by a licensed pathologist. The percentage of TGFβ-positive cells was determined based on the brown (DAB) optical density measured in the cellular membrane and cytoplasm by applying a positivity threshold of optical density = 15.

### *In vitro* CAR-T cytotoxic activity

Assessment of *in vitro* CAR-T cytolytic activity was carried out using the Agilent xCELLigence Real-Time Cell Analysis system. Impedance was measured every 10 minutes for 60 to 75 hours. Each cancer cell line was plated at optimal seeding density to result in a confluent monolayer (3e4–6.5e4 cells per well) of a 96-well eSight plate (Agilent) in a final volume of 100 μL, and the plate was kept at room temperature for 30 minutes before being loaded into the instrument. The following day, CAR-T cells were washed three times in tumor cell complete medium and then added to the wells at the indicated effector-to-target (E:T) ratio to a final well volume of 200 μL. Percent cytolysis was calculated using RTCA Software Pro (Agilent) at the indicated times after CAR-T addition.

### ELISA

Supernatants from individual wells used in the *in vitro* xCELLigence assay plates or mouse serum were collected at indicated times after the addition of CAR-T cells and diluted for each assay. Assessment of downstream cytokine secretion was carried out with either (i) a multispot V-Plex assay (Meso Scale Discovery) with the capacity to detect the proinflammatory cytokines IFNγ, TNFα, and IL2 (Meso Scale Discovery) in multiplex format or (ii) a single-plex IFNγ ELISA system (R&D Systems). For both ELISAs, the assay was run according to the manufacturer’s protocol.

### Serial antigen restimulation cytotoxicity assay

CAR-T cells and BxPC3 + CLDN18.2 were co-cultured at an E:T ratio of 1:2 and incubated for 3 to 4 days, and then cancer cell viability was measured with CellTiter-Glo reagent (Promega). BxPC3 + CLDN18.2 cells with medium alone were used as the baseline. Fresh BxPC3 + CLDN18.2 cells were plated to repeat this continuous process with CAR-T cells that maintained viability and surface CAR expression. The E:T ratio was held constant at each rechallenge in an assay as previously described (25). Culture supernatants were collected after each round of co-culture to measure IFNγ by ELISA. At the end of each antigen challenge, the percentage of surface CAR+ was determined by FC, using an anti-clone 9 scFv paratope and/or anti-TGFβRII conjugated to PE antibody. A live/dead stain (Thermo Fisher Scientific) was used to exclude dead cells.

### *In vivo* animal studies

All procedures involving animals were conducted in facilities accredited by the Association for Assessment and Accreditation of Laboratory Animal Care under the guidelines of AstraZeneca’s Institutional Animal Care and Use Committee, in accordance with the Institute for Laboratory Animal Research’s *Guide for the Care and Use of Laboratory Animals* (eighth edition) and AstraZeneca’s Bioethics Standard. Xenograft models derived from PaTu8988s HS were developed by subcutaneous injection of 10e6 cells at a 1:1 ratio of tumor cell line to Cultrex basement membrane extract (R&D Systems) mixture into the flanks of 6 to 8-week-old female NOD/SCID gamma (NSG) mice (NOD.Cg-Prkdc^scid^ Il2rg^tm1Wjl^/SzJ) or female NSG MHC class I/II double-KO mice (NOD.Cg-Prkdc^scid^ H2-K1^b-tm1Bpe^ H2-Ab1^g7-em1Mvw^ H2-D1^b-tm1Bpe^ Il2rg^tm1Wjl^/SzJ, MHC-DKO; The Jackson Laboratory) as indicated, as the use of NSG MHC class I/II double-KO mice has been shown to delay the onset of GVHD (25, 38).

PDX models derived from patients with gastric cancer, esophageal adenocarcinoma, and PDAC were chosen based on CLDN18.2 and TGFβ expression as determined by IHC analysis. Gastric cancer and esophageal adenocarcinoma *in vivo* studies were carried out in PDX models at Crown Bioscience, and PDAC PDX models were tested at AstraZeneca according to standard operating procedures. All PDAC PDX models used in this study were obtained from the AstraZeneca PDX library and had the appropriate patient consent and Institutional Review Board approval. All PDX models were developed by subcutaneous implantation of PDX fragments into the flanks of 6 to 8-week-old female NSG MHC-DKO mice (The Jackson Laboratory). To match the clinical scenario, all animals in the PDX *in vivo* studies were given CAR-T cells that had been previously frozen.

All tumors were measured at least twice per week, and tumor volume was calculated by the formula:

$$\text{Tumor}\;\text{volume}\;\left(mm^{3}\right)=\;\frac{\text{length}\;\left(\text{mm}\right)\;\times \left(\text{widt}{h\;\left(\text{mm}\right)}^{2}\right)}{2}$$

Mice were randomized when tumor volumes had reached approximately 100 to 200 mm^3^, and all CAR-T cells were administered intravenously once on day 0 at the specified dose.

For *in vivo* cytokine analysis, peripheral blood was harvested at the indicated times, and serum was separated with serum separator tubes (BD Biosciences). Cytokine levels were determined by ELISA (Meso Scale Discovery V-Plex assay) according to the manufacturer’s instructions.

### Statistical analysis

Graphs with error bars represent the mean ± SEM as indicated. The number of independent experiments and replicates are noted in the figure legend. Significant differences between analytical groups were calculated using Prism (GraphPad Software) with the indicated tests.

### Data availability

Data and supplementary data generated are available in this article. All data are available upon reasonable request to the corresponding author Allison Barrett (barretta@astrazeneca.com).

## Results

### Prevalence of CLDN18.2 in key cancer indications and examination of normal tissue expression

**Figure 1.**
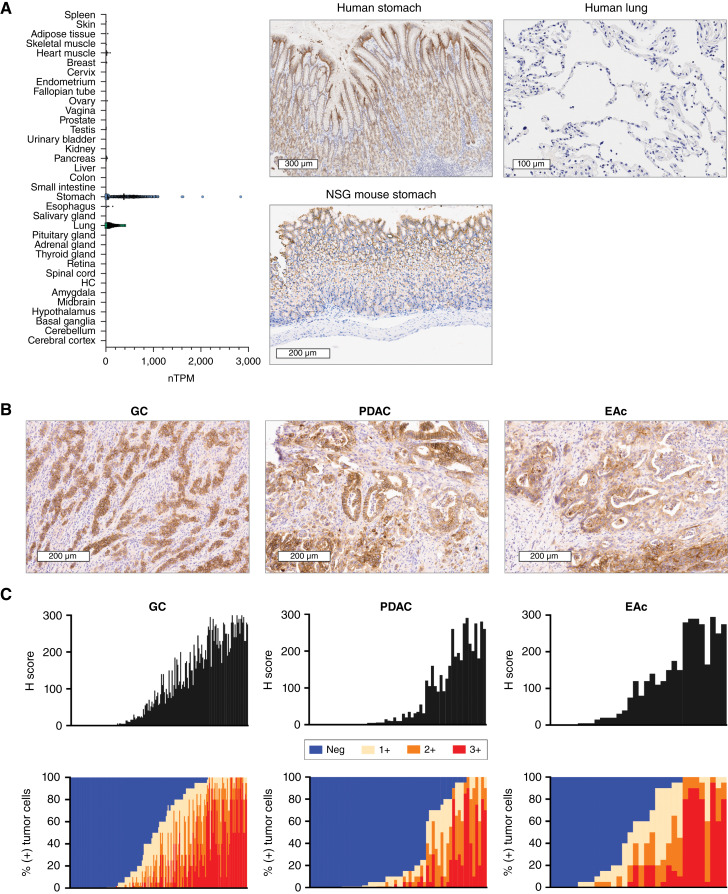
Expression and prevalence of CLDN18.2 in normal tissues and GI adenocarcinomas. **A,** Normal tissue RNA expression of *CLDN18* [represented as normalized transcripts per million (TPM); Human Protein Atlas, GTEx dataset]. HC, hippocampal formation. CLDN18.2 expression on representative images of the human stomach, NSG mouse stomach, and human lung are shown. **B,** CLDN18.2 expression on representative samples of gastric cancer (H-score = 249), PDAC (H-score = 175), and esophageal adenocarcinoma (H-score = 175). **C,** Prevalence and intensity of CLDN18.2 expression on tumor samples derived from patients with gastric cancer (*n* = 185), PDAC (*n* = 61), and esophageal adenocarcinoma (*n* = 32). Data are shown as H-scores and as the percentages of tumor cells at each level of intensity (1+, 2+, and 3+). EAc, esophageal adenocarcinoma; GC, gastric cancer.

Although CLDN18.2 is a well explored and clinically relevant target, we first sought to confirm and supplement the known literature on CLDN18.2 protein expression in normal tissues and across multiple tumor types (Fig. 1A). Using an IHC assay shown to be specific for CLDN18.2 (Supplementary Fig. S1A), we confirmed, as reported in the literature, that the predominant site of CLDN18.2 expression in normal tissue was stomach glandular epithelium (Fig. 1A), although limited CLDN18.2 expression was found in other digestive tissues, including gall bladder (data not shown). Membrane staining was observed in epithelial cells at all levels of the gastric glands and in fundic and pyloric regions. Similar expression was noted in NSG mouse stomach mucosa (Fig. 1A), which enabled murine studies to serve as models for efficacy and tolerability with murine cross-reactive reagents.

For further confirmation of the relevance of targeting CLDN18.2 in GI cancers, expression was determined by IHC and H-score calculated using formalin-fixed, paraffin-embedded TMAs encompassing more than 200 samples of patients with gastric cancer, PDAC, and esophageal adenocarcinoma that spanned cancer stages and included primary and metastatic lesions. Positive staining at any level of intensity in greater than 1% of cells was 79% for gastric cancer, 82% for PDAC, and 89% for esophageal adenocarcinoma. Representative CLDN18.2 staining (Fig. 1B) and quantitative CLDN18.2 prevalence and intensity (Fig. 1C) are shown for all three indications.

### Development of a CLDN18.2 CAR-T lead construct targeting a unique epitope with enhanced potency and *in vivo* tolerability

**Figure 2.**
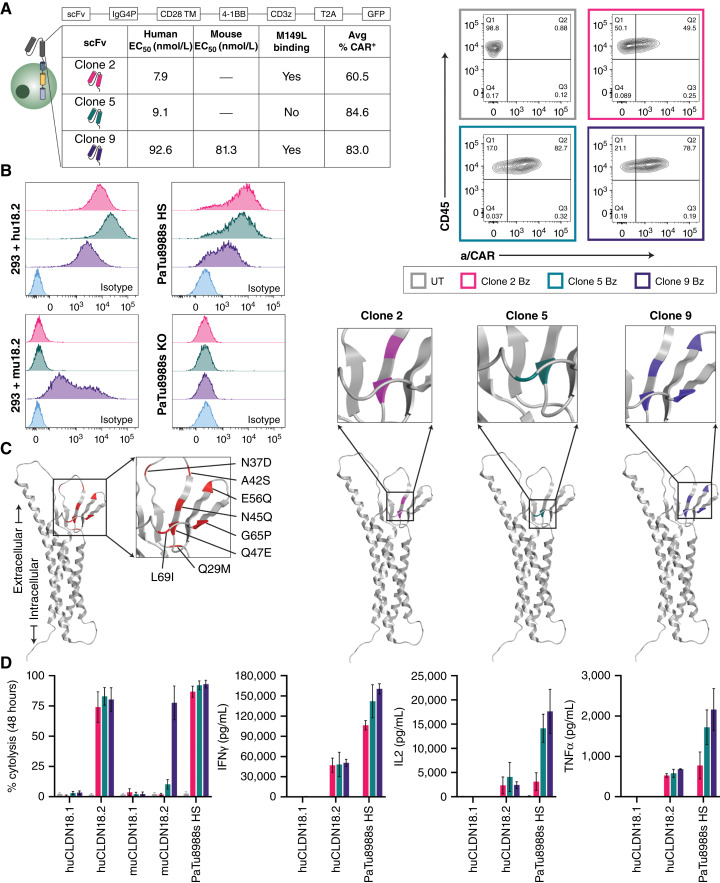
Development and evaluation of CLDN18.2-targeting CAR-T cells *in vitro*. **A,** Schematic representation of second-generation CAR-T lentivirus design, which includes a 4-1BB costimulatory domain (Bz). The table shows for each CLDN18.2-reactive clone the relative binding affinity (human and mouse), reactivity to mutant CLDN18.2 (M149L), and average transduction efficiency (CAR+, day 9) of multiple healthy donors. Representative FC plots of CAR surface expression at day 9 after lentivirus transduction were compared with UT control for a single donor. **B,** CLDN18.2 cell surface expression of various cell lines as determined by FC with 5 μg/mL CLDN18.2-reactive clones compared with nonspecific isotype antibody (R347). **C,** Epitope characterization of CLDN18.2-reactive clones. The AlphaFold structure on the far left (red) represents all sites of point mutation in HEK293 cells that vary between CLDN18.1 and CLDN18.2 in the first extracellular loop; the color-coded diagrams represent sites that influence respective clone binding. **D,** Percent cytolysis of HEK293 + huCLDN18.1, HEK293 + huCLDN18.2, HEK293 + muCLDN18.1, HEK293 + muCLDN18.2, and PaTu8988s HS cells determined by xCELLigence RTCA assay after 48 hours of co-culture with CLDN18.2 CAR-T cells at a 1:1 E:T ratio. The supernatants from the xCELLigence assay were collected at 24 hours for cytokine assessment (Meso Scale Discovery) assay. All data represent mean ± SEM of replicate experiments.

Candidate scFvs from phage selections were converted to IgG1 format for affinity and cross-reactivity assessment screening by FC. CLDN18.2 isoform reactivity and specificity were assessed by evaluating the binding of candidate clones to HEK293 cells expressing human- and mouse-derived CLDN18.1 or CLDN18.2, as well as to a PDAC-derived cell line that endogenously expresses CLDN18.2 and was enriched by FACS for a high-expressing population [PaTu8988s HS cell line; Fig. 2A and B; Supplementary Fig. S2A and S2B]. By FC, the 50% effective concentration (EC_50_) of clones 2 and 5 were similar on HEK293 + human CLDN18.2 (+huCLDN18.2) cells (7.5 and 9.1 nmol/L, respectively), whereas clone 9 had a reduced EC_50_ of 92.6 nmol/L (Fig. 2A; Supplementary Fig. S3A). Clone 9 IgG1 maintained a similar EC_50_ (81.3 nmol/L) on HEK293 + murine CLDN18.2 (+muCLDN18.2) cells (Fig. 2A; Supplementary Fig. S3A). Importantly, none of the clones bound PaTu8988s CLDN18 KO cells (Fig. 2B) or human or murine CLDN18.1-expressing cells (Supplementary Fig. S3B and S3C).

Clones 2, 5, and 9 IgGs were converted to scFv CAR-T format for functional assessments. Healthy donor T cells were transduced with the various lentivirus constructs and had an average percent CAR surface expression (mean ± SEM) of 60.5% ± 4.0%, 84.6% ± 2.0%, and 83.0% ± 2.3%, respectively, across donors (Fig. 2A). By FC, CAR-positive detection was similar when the fluorescent tag in the lentivirus vector was compared with surface detection using an anti-CAR reagent (Supplementary Fig. S3E).

Characteristics of the binding epitope, in particular its proximity to the cell membrane, have been shown to influence the potency of CAR-T–mediated cytolysis in preclinical models (39). An FC-based assay was developed to distinguish the epitopes of prospective CLDN18.2-targeted antibodies. To identify these epitopes, HEK293 variant cells were generated that each had a single point mutation (eight cell lines in total) in the eight amino acids that differentiate the CLDN18.1 and CLDN18.2 first extracellular loop (Q29M, N37D, A42S, N45Q, Q47E, E56Q, G65P, or L69I). Antibody recognition of WT and mutant CLDN18.2 cell lines was examined to denote any perturbation of the signal. Interestingly, clone 9 IgG1 demonstrated sensitivity to N45Q, Q47E, E56Q, and G65P mutations, which indicated a membrane-proximal and conformational epitope that spanned four of the eight amino acids that differ between CLDN18.2 and CLDN18.1 (Fig. 2C; Supplementary Fig. S4A and S4B). All WT sites and clone-specific sites were mapped using the AlphaFold Protein Structure Database (40, 41). The binding of clones 2, 5, and 9 was also examined using HEK293 + CLDN18.2_M149L, a naturally occurring coding single-nucleotide polymorphism that is present in a small percentage of patients with CLDN18.2-positive cancer (42). Clones 2 and 9 maintained, but clone 5 lost binding in this setting (Supplementary Fig. S3D).

Next, we examined the *in vitro* activity of the CAR-T cells in various cell lines. The CAR-T cells showed equivalent cytolysis at 48 hours when co-cultured with HEK293 + huCLDN18.2 and PaTu8988s HS, but only clone 9 showed significant cytolytic capacity in HEK293 + muCLDN18.2 (Fig. 2D). No significant activity was observed for any clone on HEK293 + huCLDN18.1 or HEK293 + muCLDN18.1 cells, indicating target specificity (Fig. 2D; Supplementary Fig. S3B and S3C). Proinflammatory cytokine production was measured after 24 hours of co-culture with antigen-positive cells, demonstrating that clone 9 CAR-T produced levels of IFNγ, IL2, and TNFα that were equivalent to or greater than those of clones 2 and 5 (Fig. 2D).

**Figure 3.**
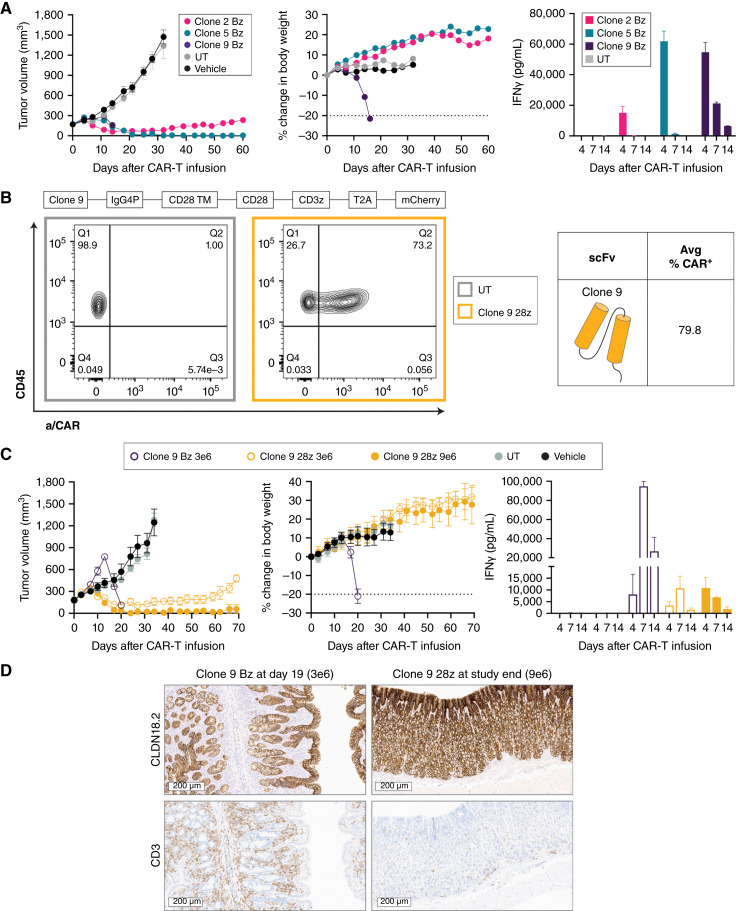
Evaluation of CLDN18.2-targeting CAR-T cells *in vivo*. **A,** NSG mice bearing PaTu8988s HS xenografts (CLDN18.2 H-score = 268) were dosed by tail vein with 9e6 CAR+ CLDN18.2 Bz CAR-T cells; total T-cell infusion number was matched across groups. Tumor volume and body weight were measured biweekly (*n* = 9). Serum levels of IFNγ were measured at 4, 7, and 14 days after infusion (*n* = 3). **B,** Schematic representation of a second-generation CAR-T design modified to replace 4-1BB with a CD28 costimulatory domain (28z). The average transduction efficiency (CAR+, day 9) of multiple healthy donors for clone 9 28z is shown. Representative FC plots of CAR surface expression at day 9 after lentivirus transduction were compared with UT control for a single donor. **C,** NSG mice bearing PaTu8988s HS xenografts were dosed as described in **A** with clone 9 CD28z or Bz CAR-T (*n* = 6) at indicated doses. Serum levels of IFNγ were measured at 4, 7, and 14 days after infusion (*n* = 3). **D,** Representative images of CLDN18.2 (top row) and CD3 (bottom row) staining in the stomachs of mice dosed with clone 9 CAR-T cells from **C** at indicated time points. All data represent mean ± SEM of replicate experiments or animals.

To determine antitumor activity *in vivo*, 9e6 4-1BB CD3z (Bz) CAR-T cells or untransduced donor-matched control T cells (UT) were administered via a single infusion in the tail veins of NSG mice bearing PaTu8988s HS xenografts. All CAR clones showed strong antitumor activity at this dose, although tumor outgrowth was observed by day 60 in mice treated with clone 2 Bz. Clone 5 Bz CAR-T cells showed a durable complete response until the end of the study (Fig. 3A). The murine cross-reactive clone 9 Bz CAR-T cells exhibited an initial tumor response that was followed by rapid body weight loss requiring euthanasia. Owing to the strong expression of CLDN18.2 in mouse gastric tissue, coupled with the murine cross-reactivity of clone 9 (Figs. 1A and 2A), we concluded that the weight loss could be a result of on-target, off-tumor (OTOT) gastric toxicity. Interestingly, serum cytokine analysis further established that clone 9 Bz CAR-T–treated mice exhibited more sustained IFNγ than either clone 2 or clone 5 CAR-T cells (Fig. 3A), likewise implicating a more sustained activation state. The clones were also examined as first-generation CAR-T cells, lacking a costimulatory domain, which indicated that the best antitumor activity followed dosing with clone 9 and without signs of OTOT activity, as body weight was stable (Supplementary Fig. S5A). It has been reported that modification of the number of immunoreceptor tyrosine-based activation motifs in the CAR-T CD3z chain can reduce CAR-T cell signal strength and effector functions (43, 44); however, this approach in the context of clone 9 4-1BB CAR-T delayed but did not abrogate the onset of OTOT activity in NSG mice bearing PaTu8988s HS tumors (Supplementary Fig. S5B). Altogether, despite reduced binding affinity as determined by EC_50_, clone 9 maintained the highest degree of functional potency.

In light of these results, we next wanted to determine whether the efficacy-to-tolerability window could be further optimized by comparing clone 9 containing a CD28 (28z) *versus* a Bz intracellular domain. After transduction, surface CAR+ was 79.8% ± 0.9% for clone 9 28z, in line with that observed with clone 9 Bz (Fig. 3B) and with expression determined by fluorescent tag (Supplementary Fig. S3F). Reduction of clone 9 Bz CAR-T dose maintained antitumor efficacy but also was not tolerated (Fig. 3C). On the contrary, 9e6 clone 9 28z CAR-T cells produced long-lasting tumor control and 3e6 produced extended tumor growth delay, and both doses were tolerated in animals (Fig. 3C). Serum cytokine analysis showed that the reduced dose of 3e6 Bz CAR-T cells still produced and sustained high IFNγ release compared with 28z CAR-T cells (Fig. 3C). Pathology review of mouse stomach from clone 9 28z-treated animals at study endpoint showed maintenance of CLDN18.2 expression and minimal infiltration in both glandular and nonglandular regions of CD3 cells in line with that to be expected of physiologic circulation, with no noted damage to the mucosal epithelium (Fig. 3D). Conversely, pathology review confirmed damage to the gastric epithelium and significant CD3 cell infiltration in the gastric mucosa of animals treated with 3e6 clone 9 Bz CAR-T cells at time of tissue harvest (Fig. 3D). On the basis of these results, a second-generation CAR-T with the clone 9 scFv and CD28 costimulatory domain was selected for further preclinical evaluation.

### Effect of adding TGFβ armoring on the efficacy of lead CAR-T in key indications

**Figure 4.**
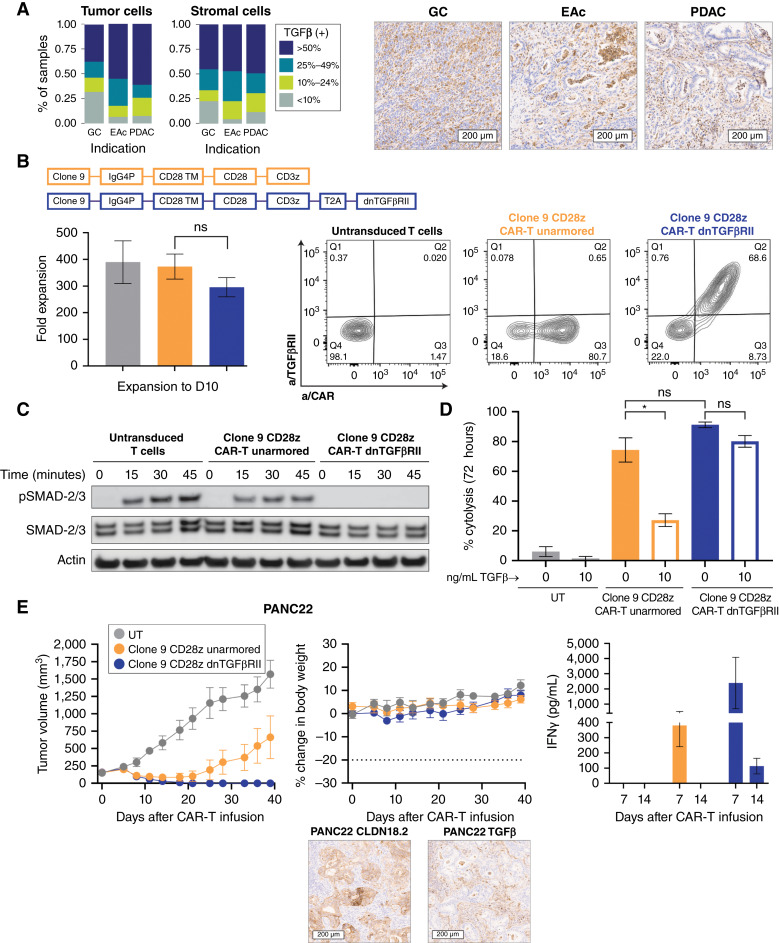
Rationale for selection of TGFβ armoring and proof of mechanism *in vitro* and *in vivo*. **A,** Quantitative analysis of intensity and prevalence and representative images of TGFβ staining in a subset of patient tumor samples [gastric cancer (GC), *n* = 130; esophageal adenocarcinoma (EAc), *n* = 15; PDAC, *n* = 71]. Data represent the pooled scores of tumor and stromal compartments. **B,** Schematic representation of second-generation CAR-T lentivirus design, including an IgG4P hinge, CD28 transmembrane, CD28 costimulatory domain, and CD3z (unarmored CAR-T cells) or additional T2A self-cleaving peptide and dnTGFβRII (armored CAR-T cells). Average fold expansion across multiple healthy donors is shown. There was no significant difference in expansion across groups (one-way ANOVA). Representative FC plots show CAR and TGFβRII surface expression at day 10 after lentivirus transduction compared with UT control. **C,** FACS-purified, serum-starved CAR-T cells were stimulated with 1 ng/mL rhTGFβ for various time periods. Western blotting was used to determine protein levels of p-SMAD2/3 and total SMAD2/3; β-actin was used as the loading control. **D,** Percent cytolysis of BxPC3 + CLDN18.2 cells as determined by xCELLigence RTCA assay after 72 hours of co-culture at a 1:1 ratio with CLDN18.2 CAR-T cells in the presence or absence of 10 ng/mL rhTGFβ. Results were analyzed by using paired *t* tests. **E,** NSG mice bearing pancreatic PDX (PANC22, H-score = 225, TGFβ intermediate) were dosed by tail vein with 3e6 CAR+ unarmored or dnTGFβRII CLDN18.2 CAR-T cells; the total T-cell infusion number was matched across groups. Tumor volume and body weight were measured biweekly (*n* = 5). Serum levels of IFNγ were measured at 7 and 14 days after infusion (*n* = 3). Representative images of CLDN18.2 and TGFβ staining IHC expression (20× scan) are shown. All data represent mean ± SEM of replicate experiments or animals.

To confirm the reported overabundance of TGFβ in GI tract TMEs (45), a subsequent slide of TMAs previously profiled for CLDN18.2 expression was stained to examine protein levels of TGFβ by IHC (Fig. 4A). A considerable percentage of each sample on the TMAs exhibited high stromal and tumor cell TGFβ staining, leading us to explore the use of dnTGFβRII armoring in these cancer indications.

Clone 9 28z CAR-T cells were generated with or without dnTGFβRII. Cells were then cultured for 9 to 10 days with an average 300 to 400-fold expansion across groups (Fig. 4B). Surface expression of CAR and dnTGFβRII was confirmed by FC, and the majority of dnTGFβRII CAR-T cells stained positive for both (Fig. 4B).

To demonstrate proof of the mechanism of the dnTGFβRII, a pure CAR+ population was sorted by FACS, serum starved for 24 hours and then stimulated with 1 ng/mL recombinant human TGFβ (rhTGFβ). Downstream TGFβ signaling capacity was examined by monitoring phosphorylation of SMAD2/3 at 15, 30, and 45 minutes after co-culture. Phospho-SMAD2/3 (p-SMAD2/3) was observed in both UT cells and unarmored CAR-T cells, but no p-SMAD2/3 was detected up to 45 minutes after rhTGFβ exposure in dnTGFβRII CAR-T cells (Fig. 4C).

Additional *in vitro* proof of the mechanism of dnTGFβRII armoring was demonstrated by monitoring BxPC3 + CLDN18.2 cell lysis after 72 hours of co-culture with unarmored CAR-T cells or dnTGFβRII CAR-T cells in the presence or absence of 10 ng/mL rhTGFβ. In the medium alone, both unarmored and dnTGFβRII CAR-T cells efficiently lysed CLDN18.2-expressing cells, with no significant difference in potency. In contrast, the addition of 10 ng/mL rhTGFβ significantly reduced tumor cell lysis of the unarmored CAR-T cells (Fig. 4D) with no significant effect on the cytolytic capacity of dnTGFβRII CAR-T cells. Including 10 ng/mL rhTGFβ also reduced the ability of unarmored clone 9 to maintain serial rounds of BxPC3 + CLDN18.2 cell lysis, in contrast to the maintained activity of dnTGFβRII CAR-T (Supplementary Fig. S6A).

To examine how armoring against TGFβ translated *in vivo*, a PDX model of PDAC was administered 3e6 UT, unarmored CAR-T cells, or dnTGFβRII CAR-T cells. This PDX model expressed a moderate level of both CLDN18.2 and TGFβ, as determined by IHC (Fig. 4E). IHC examination of phosphorylated SMAD2 in representative xenograft models was co-localized with regions that stained positive for TGFβ1, likely indicating active TGFβ signaling (data not shown). Although an initial tumor response was observed, tumors in animals administered unarmored CAR-T cells showed outgrowth beyond day 20 (Fig. 4E). In contrast, dnTGFβRII CAR-T cells produced durable and complete tumor regression in all mice. Serum cytokine analysis demonstrated that dnTGFβRII CAR-T cells produced more IFNγ than unarmored CAR-T cells but, unlike Bz CAR, was not associated with OTOT toxicity (Fig. 4E). Thus, dnTGFβRII armoring improved the activity of clone 9 28z CAR-T in a patient-derived, TGFβ-positive tumor model but did not result in compromised tolerability or decreased therapeutic index.

### Optimization of CAR-T functional longevity via short manufacturing

We then sought to determine whether further enhancement of the CAR-T product could be achieved through the use of an optimized manufacturing process. Isolated donor T cells were split to control for donor variability and then manufactured with either a traditional protocol (expansion for 9–12 days in 300-IU/mL IL2) or a modified protocol, which we named the “stem cell–like armored responsive T-cell platform” (STAR-T) process. With STAR-T, cells were propagated with a reduced concentration of IL2 and with the addition of IL21 in a shortened (4-day) expansion process. CAR-T cells manufactured via the STAR-T process and with the inclusion of dnTGFβRII were thus named “AZD6422.”

**Figure 5.**
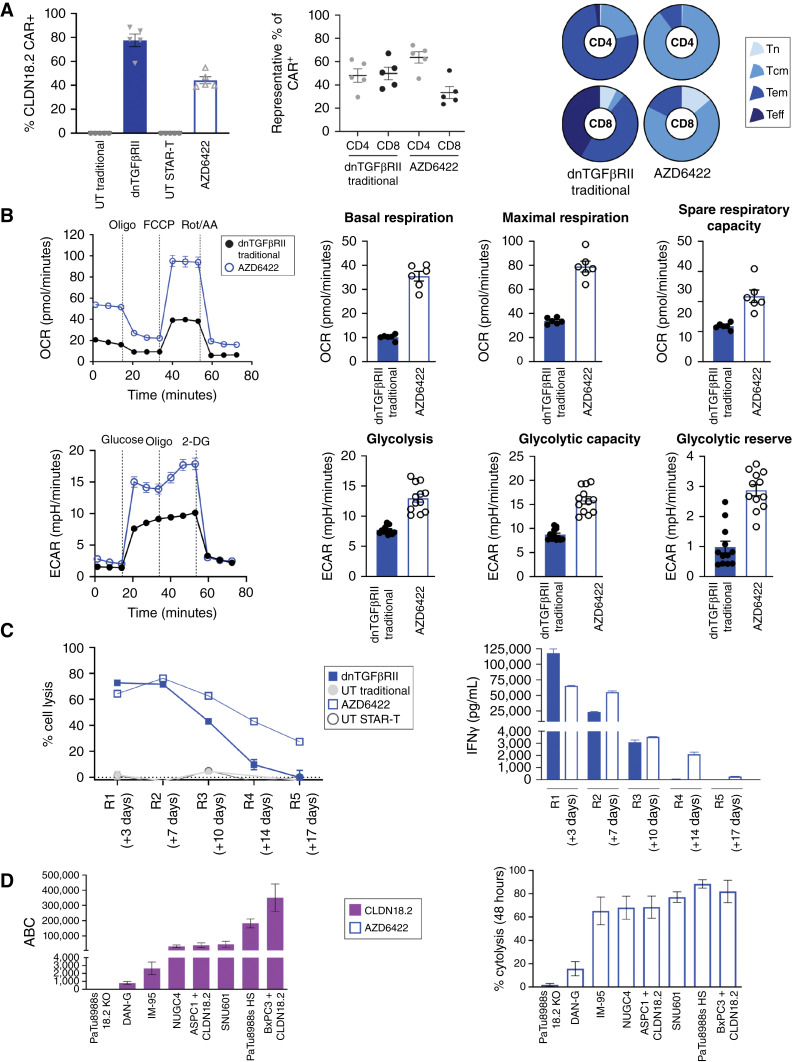
Optimized manufacturing protocol, STAR-T, for the generation of the CAR-T product. **A,** Baseline characteristics of donor-matched dnTGFβRII CAR-T cells with traditional manufacture (day 10) vs. AZD6422 (day 4), including CAR+ expression, percent CD4 and CD8 expression, and T-cell phenotypic status as determined by cell surface expression of CCR7 and CD45RO. Results are shown for naïve (CCR7+/CD45RO^−^, Tn), central memory (CCR7^+^/CD45RO^+^, Tcm), effector memory (CCR7^−^/CD45RO^+^, Tem), and effector (CCR7^−^/CD45RO^−^, Teff) cells. Data are shown as mean ± SEM of representative donors. **B,** Comparison of bioenergetic profiles of traditionally manufactured dnTGFβRII CAR-T cells *vs.* AZD6422. Spare respiratory capacity was determined as the differential between basal and maximum respiration. 2-DG, 2-deoxy-D-glucose; ECAR, extracellular acidification rate; FCCP, carbonyl cyanide p-trifluoro-methoxyphenyl hydrazone; OCR, oxygen consumption rate; Oligo, oligomycin; Rot/AA, rotenone and antimycin A. **C,** Serial restimulation assay to examine cytotoxicity and persistence of dnTGFβRII CAR-T cells and AZD6422. CAR-T cells were co-cultured at a ratio of 1:2 with BXPC3 + CLDN18.2, tumor lysis was measured every 3 to 4 days, and IFNγ was profiled at 24 hours after each new co-culture. Representative of multiple donors. **D,** Results of quantitative FC to determine cell surface expression of CLDN18.2 across multiple cancer cell lines. Percent cytolysis was determined by xCELLigence RTCA assay after 48 hours of co-culture with AZD6422 at an E:T ratio of 1:1. Data represent mean ± SEM of replicate experiments.

Surface expression of the CAR was reduced for AZD6422, with an average of 44.21% CAR+ across donors, as compared with 77.5% for traditionally manufactured dnTGFβRII CAR-T cells (Fig. 5A). AZD6422 contained a greater percentage of CD4 cells and T memory stem cells/naïve and central memory cells than traditionally manufactured dnTGFβRII, as determined by FC (Fig. 5A). Furthermore, metabolic analysis of the CAR-T products revealed that, compared with traditionally manufactured cells, AZD6422 had increased basal respiration, maximal respiration, and spare respiratory capacity, as well as increased glycolysis and glycolytic capacity (Fig. 5B).

To determine the impact of these observed differences in T-cell phenotype and metabolism on CAR-T functionality, matched donor CAR-T cells from the two processes were tested in a serial restimulation assay. CAR-T cells were co-cultured with fresh BxPC3 + CLDN18.2 cells every 3 to 4 days at an E:T ratio of 1:2 in a culture medium containing 10 ng/mL rhTGFβ. Although similar potency was observed for the first two rounds of tumor cell lysis, in the later rounds of the assay AZD6422 maintained superior tumor cell lysis activity and cytokine production beyond that of the traditionally manufactured dnTGFβRII CAR-T cells (Fig. 5C).

We next examined the efficacy of AZD6422 CAR-T tumor cell lysis across a panel of cancer cell lines with various levels of CLDN18.2 expression, as determined by quantitative FC (Fig. 5D). Cancer cell lines and AZD6422 CAR-T cells were co-cultured (1:1), and at 48 hours percent cytolysis was calculated for each cell line. AZD6422 maintained specific and potent activity across a range of CLDN18.2-expressing cell lines (Fig. 5D) and demonstrated no cytolytic activity in a CLDN18 KO PaTu8988s cell line (Supplementary Fig. S2C).

### Efficacy and tolerability of AZD6422 in representative PDX models across multiple GI cancer indications

**Figure 6.**
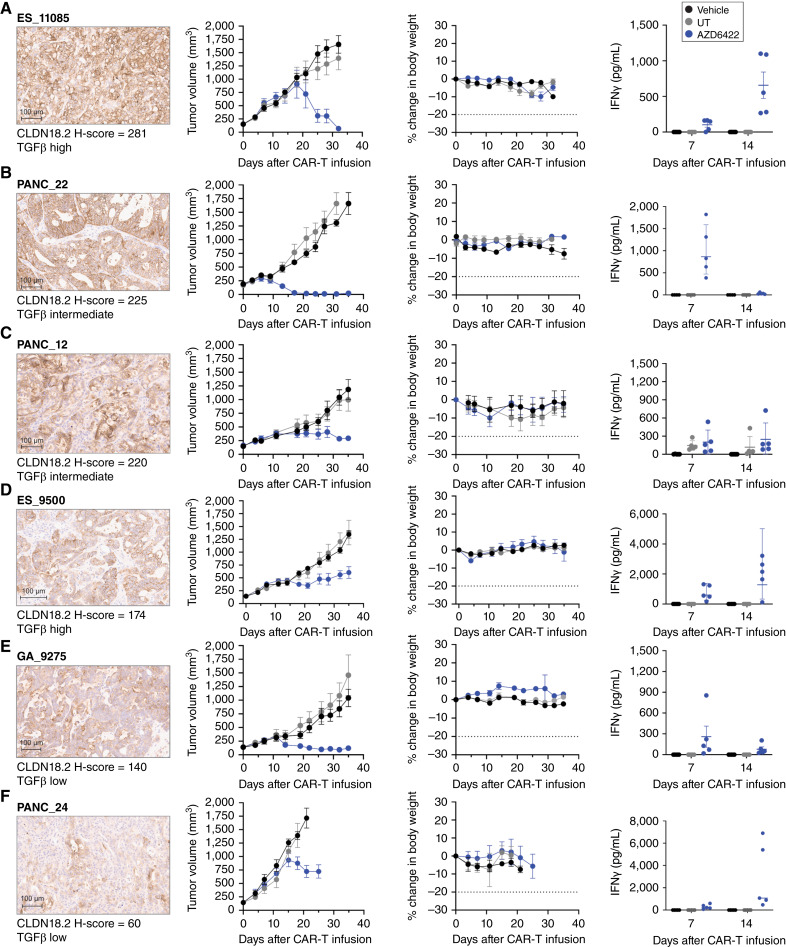
*In vivo* antitumor activity of AZD6422 in PDX models of gastric cancer, PDAC, and esophageal adenocarcinoma. Activity and tolerability of AZD6422 are shown in various PDX models of esophageal adenocarcinoma (**A**, ES11085; **D**, ES_9500), gastric cancer (**E**, GA_9275), and PDAC (**B**, PANC_22; **C**, PANC_12; **F**, PANC_24). Each model was selected to represent a range of CLDN18.2 (shown at 10× scan) and TGFβ expression. NSG MHC-DKO mice received a single tail-vein infusion of 1e6 AZD6422, donor-matched UT, or vehicle when the average tumor volume reached 150 mm^3^. Tumor volumes and body weights were measured biweekly until study completion on day 35, and blood was collected for cytokine analysis on days 7 and 14. Data are shown as mean ± SEM (*n* = 5).

The efficacy of AZD6422 was subsequently examined in a panel of PDXs representing a variety of solid tumor adenocarcinomas and a range of CLDN18.2 and TGFβ expression. AZD6422 demonstrated efficacy and tolerability across all PDX models at a modest dose of 1e6 CAR+ cells with detectible IFNγ release at varied times post–CAR-T infusion (Fig. 6). Tumor response correlated with CLDN18.2 but not TGFβ expression as a growth delay rather than regression was observed in models with lower CLDN18.2 H-scores (Fig. 6; Supplementary Fig. S7). Importantly, ES_11085 and ES_9500 were derived from patients with esophageal cancer in whom prior standard-of-care chemotherapy had failed (huBase; Crown Bioscience); AZD6422 may therefore have potential in tumors previously exposed to prior lines of therapy, including chemotherapeutic agents. Overall, these *in vivo* studies demonstrated that AZD6422 demonstrated efficacy and tolerability in representative patient-derived models of challenging clinical solid tumor indications.

## Discussion

Systemic chemotherapy remains the standard of care for advanced and metastatic gastric and gastroesophageal junction cancers, but immuno-oncology and targeted therapy approaches are beginning to show promise (46–48). Continued advancement of next-generation approaches is thus needed to improve outcomes in these indications. Molecularly driven therapeutic approaches, including antibody-based approaches targeting HER2 and CLDN18.2, have been gaining momentum in the gastric cancer therapeutic space (49, 50). The phase III GLOW and SPOTLIGHT clinical trials demonstrated that the combination of zolbetuximab, a CLDN18.2-targeting mAb, with chemotherapy significantly extended PFS in patients with CLDN18.2-positive, HER2-negative advanced GI cancer and demonstrated a manageable safety profile in support of CLDN18.2 as a targetable tumor-associated antigen (16, 17). Furthermore, clinical results from a phase I trial of CT041, a CLDN18.2-targeted CAR-T, in heavily pretreated patients with GI cancer demonstrated promising efficacy and an acceptable safety margin; however, median persistence of CT041 was 28 days after the first infusion (18). Here, we present the preclinical development of AZD6422, a clinical-stage CLDN18.2-targeting CAR-T augmented with defensive armoring against TGFβ, which is abundant and suppressive within the GI TME, together with an optimized manufacturing protocol to generate cells for infusion that present with a favorable metabolic phenotype.

Many factors can influence CAR-T activity, including epitope proximity to the cell surface, CAR-T spacer length and flexibility, and CAR-T functional avidity, as determined by binding affinity of the scFv, CAR surface expression, and antigen density (44, 51–54). Ideal epitope proximity, however, is not universal, and there are disparate examples of CAR-T and epitope pairs that favor either distal epitopes or membrane-proximal epitopes for facilitating optimal CAR-T activation (55). FC-based epitope mapping revealed that the scFv component of AZD6422, clone 9, recognized a membrane-proximal and conformational epitope that spanned four of the eight amino acids that differ between CLDN18.1 and CLDN18.2. Furthermore, despite having a reduced binding affinity (EC_50_) relative to other clones, clone 9 maintained equivalent potency and produced an equal or greater amount of IFNγ upon antigen exposure. In the context of solid tumors, Mao and colleagues (53) have proposed that there may be an ideal, moderate antigen-binding domain affinity relative to clinical response. They speculate that high antigen-binding domain affinity may be detrimental as it becomes difficult for CAR-T cells to dissociate from dying tumor cells in order to continue their tumor cell killing. In addition, these investigators suggest that over-activation may drive CAR-T cells to exhaustion or to activation-induced cell death and that a “fast-on, fast-off” mode of engagement may be ideal for CAR-T cells in solid tumors surrounded by antigen-expressing cells (53). Indeed, clone 9 outperformed clones with higher affinity in a serial restimulation assay with a matched CAR-T design (Bz; data not shown). Thus, its unique binding epitope and reduced EC_50_ may favor clone 9 within the context of solid tumors.

To date, most second-generation CAR-T cells that have reached the clinical stage use for co-stimulation either CD28 or 4-1BB, both of which can impart distinct attributes to the final product. For instance, the choice of a costimulatory domain can impact CAR-T metabolism because CD28z cells rely heavily on aerobic glycolysis, whereas 4-1BBz cells prefer fatty acid oxidative metabolism (56). The TME of pancreatic and gastroesophageal cancers often contain hypoxic regions that favor glycolytic metabolism, and thus the choice of CD28 co-stimulation may provide an advantage in hypoxic solid tumors (39, 57–60). It has also been reported that the selection of CD28 co-stimulation endows both CD19 and HER2 CAR-T cells with superior activity at lower antigen receptor densities (44). Notably, however, the hinge and transmembrane also differed between products in these studies, thereby confounding conclusions because these components are also critical to CAR-T functionality (44, 54). Muller and colleagues (61) reported that the use of the CD28 transmembrane domain promotes heterodimerization with endogenous CD28 on T cells, which may induce stronger signal transduction to account for this increased activity in low-antigen settings. Therefore, CD28 co-stimulation may also be important in the translation of CAR-T activity to potentially heterogeneous antigen-expressing solid tumors. In our studies, we found that AZD6422 CAR-T cells maintained activity across a range of cancer cell lines with various receptor densities of CLDN18.2.

The potential for OTOT CAR-T activity is a challenge that should be considered for CLDN18.2-targeted therapies because of CLDN18.2 expression on normal gastric mucosal cells (62). Indeed, gastric-associated on-target toxicities were more common in zolbetuximab-treated patients than in those given placebo, although the adverse effects were manageable (16, 17). GI-related adverse events were reported in 8.2% of patients after the administration of CT041 but were manageable (18). The normal tissue expression profile of CLDN18.2 is maintained in gastric epithelium across animal species and murine homology is 89%, which is an important consideration in the use of murine models in safety assessments (63). Preclinical data on the CT041 product, which also possesses a CD28 costimulatory domain, showed tolerability in mice, which was attributed to the potential exclusion of normal tissue and the capacity for rapid tissue repair (64). Importantly, our results showed that clone 9 maintained species cross-reactivity and EC_50_ values that were comparable with humans, thus enabling preclinical mouse modeling assessments of clone 9 CAR-T signaling and functionality. In our studies, clone 9 was well tolerated and efficacious as a 28z CAR-T, though not tolerated as a Bz CAR-T.

A number of hypotheses could account for these results. An extensive review of preclinical and clinical data comparing CAR-T with 4-1BB or CD28 co-stimulation showed that although neither was preclinically superior in antitumor activity, most studies showed extended persistence of CAR-T cells incorporating a 4-1BB costimulatory domain in murine studies (65), which may be of importance for CAR-T targeting antigens with OTOT liabilities. Published reports have highlighted distinct metabolic profiles between CAR-T containing either a CD28 or 4-1BB costimulatory domain, with enhanced mitochondrial biogenesis in 4-1BB CAR-T cells contributing to enhanced persistence (56). To this end, we hypothesize that the extended period of IFNγ release observed in mice infused with an equivalent dose and surface CAR+ of clone 9 Bz, as compared with clone 9 28z, may indicate that OTOT toxicity was due to extended persistence and proliferation of clone 9 Bz, which may also be visualized by the tumor swelling prior to tumor clearance observed (Fig. 3C). Future work will delve into the mechanisms underlying our findings; however, as CD28 was found to be safe and efficacious in the context of CLDN18.2, this was the design used in the development of AZD6422.

GI tumors have a highly immunosuppressive TME that is partly attributed to inhibitory cytokines, including TGFβ secreted by suppressive regulatory T cells, myeloid-derived suppressor cells, and tumor-associated macrophages, which can result in reduced effector function of T cells and CAR-T cells (19). TGFβ is also associated with enhanced epithelial-to-mesenchymal transition, immune evasion, and fibrosis in PDAC (66). In preclinical studies, expression of dnTGFβRII on CAR-T cells has been reported to enhance proliferative capacity, cytokine secretion, and antitumor efficacy (24). In accordance with published data, we observed a high abundance of TGFβ in tumor and stromal compartments of gastric cancer, esophageal adenocarcinoma, and PDAC, and the inclusion of this armoring approach increased CAR-T potency *in vitro* and *in vivo* against model systems of these indications.

Longer *ex vivo* expansion of the CAR-T product has been linked to the production of exhausted cells. For this reason, shortening the expansion time may improve CAR-T quality by producing a less differentiated product and benefit patients by also reducing the time from apheresis to reinfusion, potentially shortening the duration of disease progression during manufacture (27, 32, 67). The CT041 clinical study examined the correlation of a preinfusion T-cell phenotype with clinical response as an exploratory endpoint, showing that reduced numbers of terminally differentiated effector cells correlated to longer PFS (68). The preclinical data presented here confirmed that optimized manufacture and favorable phenotype of AZD6422 CAR-T cells enabled extended T-cell activity in the presence of soluble TGFβ in a serial restimulation assay, as well as antitumor responses at a modest dose of AZD6422 in several relevant PDX models.

Altogether, the results shown here demonstrate that rational selection of endodomain and armoring, as well as enhanced manufacturing, can enhance the therapeutic potential of AZD6422. The results of preclinical efficacy, tolerability, and persistence studies warrant its continued clinical testing in patients with GI tumors.

## Supplementary Material

Supplementary Data1 Final supplementary data.
