# Supplementary material for: Preclinical Evaluation of AZD6422, an Armored Chimeric Antigen Receptor T Cell Targeting CLDN18.2 in Gastric, Pancreatic, and Esophageal Cancers
Source: Clin Cancer Res. 2024 Sep 25;30(23):5413–29. doi: 10.1158/1078-0432.CCR-24-1853 (PMC11609629; doi:10.1158/1078-0432.CCR-24-1853)
Supplement: Supplementary Data1 — Final supplementary data. [file ccr-24-1853_supplementary_data1_suppds1.pdf]

Supplemental Fig. 1

S1A

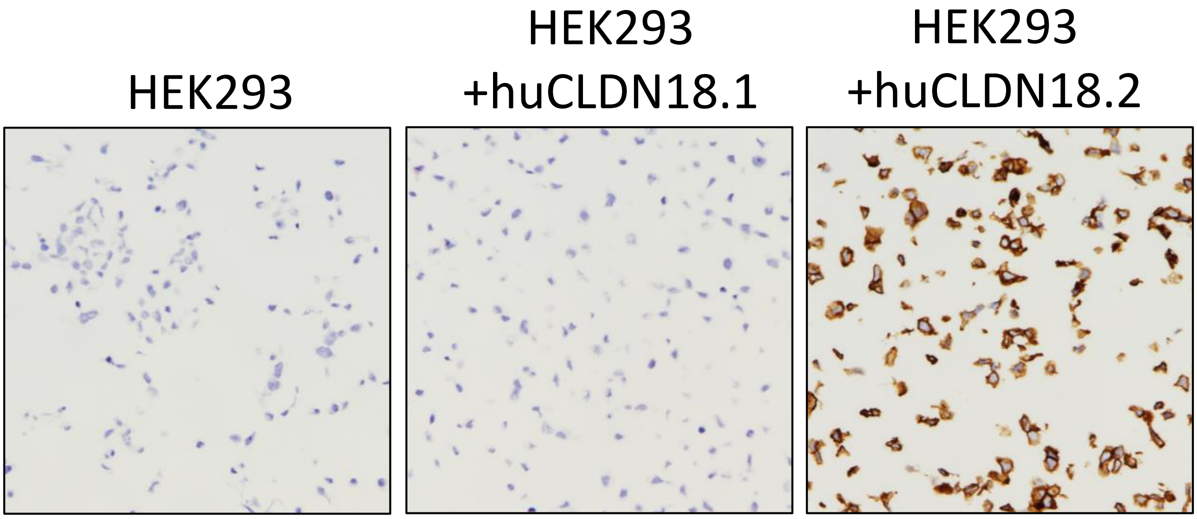

Supplemental Fig. 1

S1A: Specificity of CLDN18.2 IHC assay. HEK293 cells were engineered to express CLDN18.1 and 18.2, then cell pellets were formalin-fixed and paraffin-embedded for IHC analysis. The primary antibody used for IHC (clone EPR19202, Abcam) yielded positive staining for CLDN18.2 but not for CLDN18.1, demonstrating specificity.

Supplemental Fig. 2

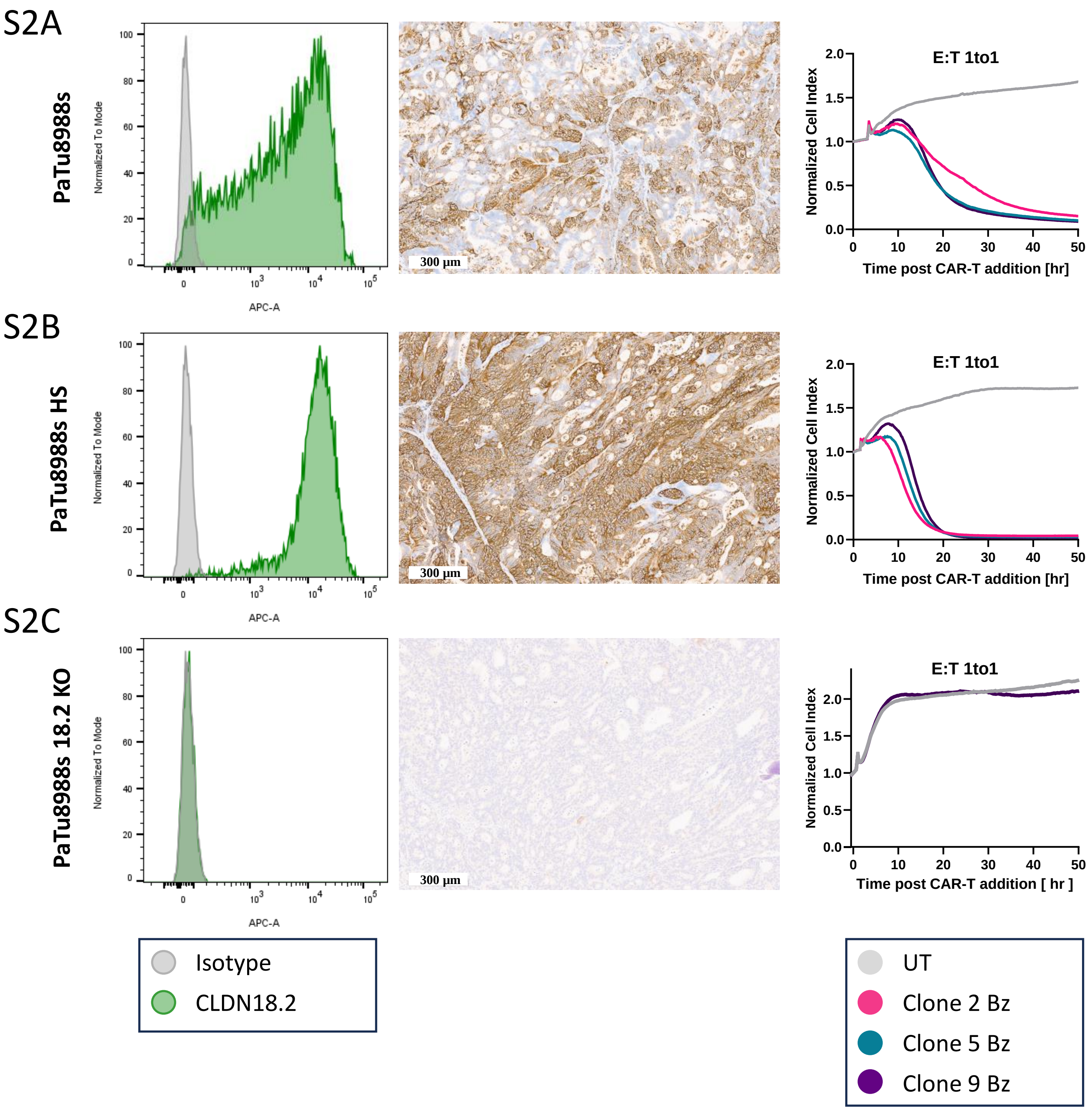

Supplemental Fig. 2

S2A: CLDN18.2 cell surface expression of PaTu8988s unsorted by FC compared to nonspecific isotype antibody (R347), IHC of PaTu8988s xenograft, 10X scan. PaTu8988s unsorted were cocultured with CLDN18.2 reactive clones at 1:1 ratio and cell lysis was monitored real-time xCelligence RTCA assay (Agilent) for 50 hours.

S2B: CLDN18.2 cell surface expression of PaTu8988s HS by FC compared to nonspecific isotype antibody (R347), IHC of PaTu8988s HS xenograft, 10X scan. PaTu8988s HS were cocultured with CLDN18.2 reactive clones at 1:1 ratio and cell lysis was monitored real-time xCelligence RTCA assay (Agilent) for 50 hours.

S2C: CLDN18.2 cell surface expression of PaTu8988s 18.2 KO by FC compared to nonspecific isotype antibody (R347), IHC of PaTu8988s 18.2 KO xenograft, 10X scan. PaTu8988s 18.2 KO were cocultured with CLDN18.2 reactive clones at 1:1 ratio and cell lysis was monitored real-time xCelligence RTCA assay (Agilent) for 50 hours.

Supplemental Fig. 3

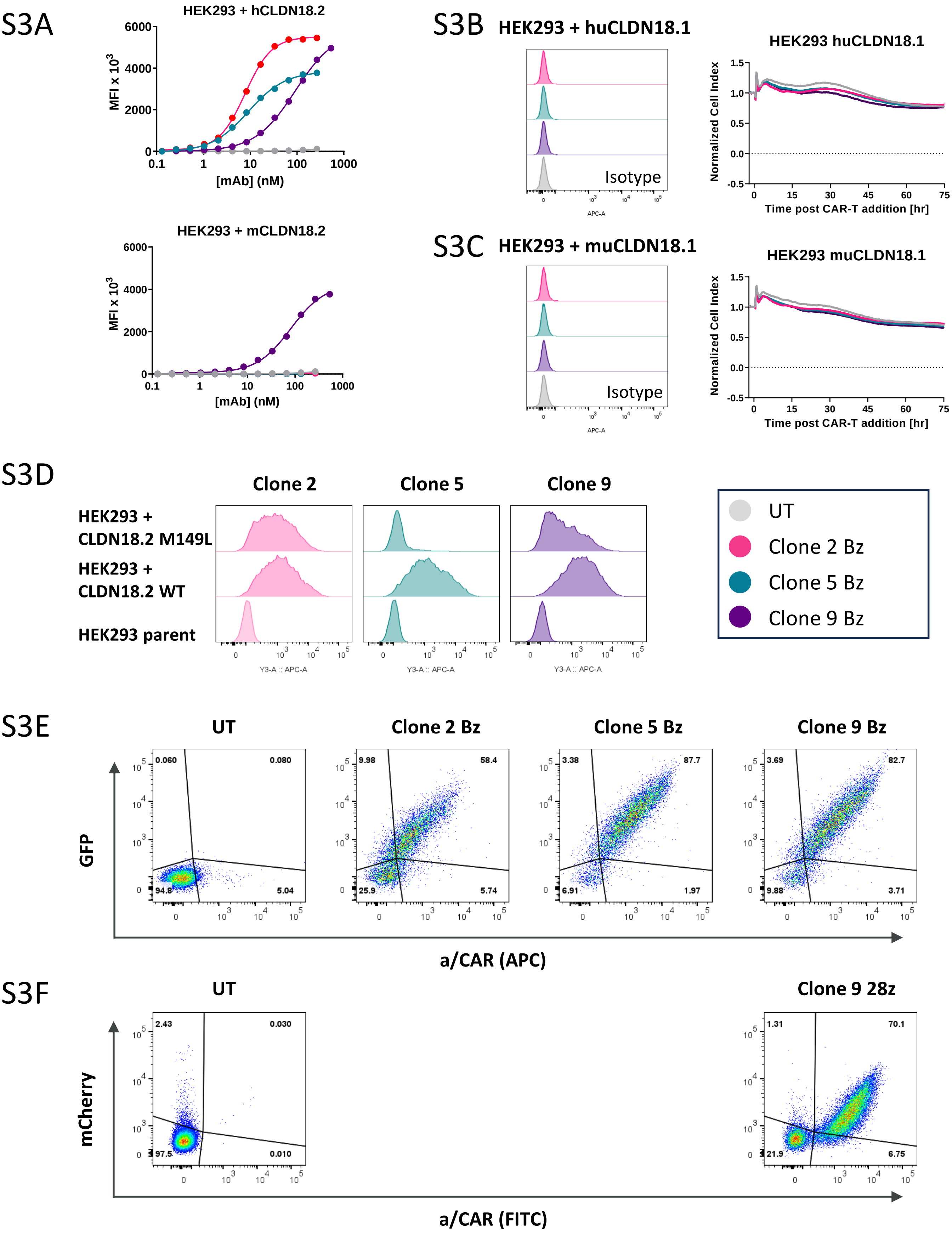

Supplemental Fig. 3

S3A: Determination of relative binding affinity of each CLDN18.2 reactive clone using HEK293 cells expressing human CLDN18.2 and murine CLDN18.2. Also included is nonspecific isotype antibody (R347).

S3B: Lack of binding of CLDN18.2 reactive clones to HEK293+human CLDN18.1 as determined by FC, also included is nonspecific isotype antibody (R347). HEK293+human CLDN18.1 were cocultured with CLDN18.2 reactive clones at 1:1 ratio and cell lysis was monitored real-time xCelligence RTCA assay (Agilent) for 75 hours.

S3C: Lack of binding of CLDN18.2 reactive clones to HEK293+murine CLDN18.1 as determined by FC, also included is nonspecific isotype antibody (R347). HEK293+murine CLDN18.1 were cocultured with CLDN18.2 reactive clones at 1:1 ratio and cell lysis was monitored real-time xCelligence RTCA assay (Agilent) for 75 hours.

S3D: HEK293 parent, HEK293+CLDN18.2 (wild type, WT) and HEK293+CLDN18.2 engineered to have the M149L SNP cell lines were examined for surface binding of CLDN18.2 reactive clones by FC.

S3E: CLDN18.2 Bz CAR-T lentiviruses included a GFP tag, comparison of surface CAR+ detection with an anti-CAR antibody reagent versus GFP are shown. UT used as control

S3F: CLDN18.2 clone 9 28z CAR-T lentivirus included an mCherry tag, comparison of surface CAR+ detection with an anti-CAR antibody reagent versus mCherry is shown. UT used as control

Supplemental Fig. 4

S4A

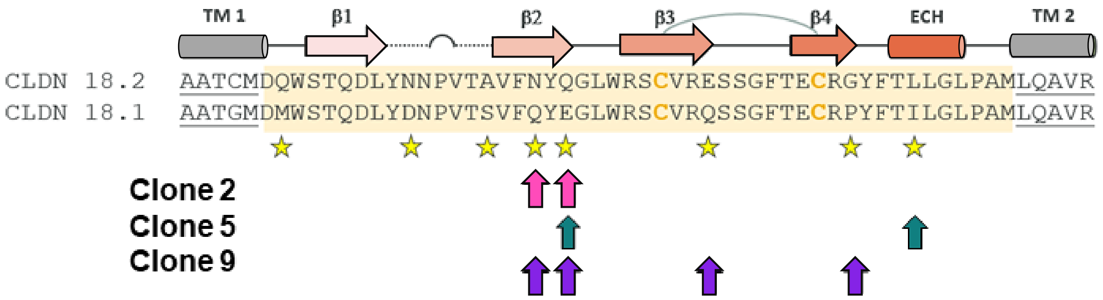

S4B

mAb	CLDN18.2 Q29M	CLDN18.2 N37D	CLDN18.2 A42S	CLDN18.2 N45Q	CLDN18.2 Q47E	CLDN18.2 E56Q	CLDN18.2 G65P	CLDN18.2 L69I
Clone 2	NE	NE	NE	Ab	Ab	NE	NE	NE
Clone 5	NE	NE	NE	NE	Ab	NE	NE	Ab
Clone 9	NE	NE	NE	INF	Ab	INF	INF	NE

Supplemental Fig. 4

S4A: Epitope characterization of select CLDN18.2-reactive clones. Differential binding to HEK293 cells expressing CLDN18.2 wild-type and CLDN18.2 variants that differ by 1 amino acid each, present in CLDN18.1. Stars represent site of point mutation in relation to CLDN18.2 extracellular structure. Arrows represent sites that influence clone binding.

S4B: Results of S4A are reported as no effect on binding (NE), influenced binding (INF) and abolished binding (Ab) to a given variant, where residues responsible for influence or loss of binding indicate residues responsible for CLDN18.2 isoform specificity.

Supplemental Fig. 5

S5A

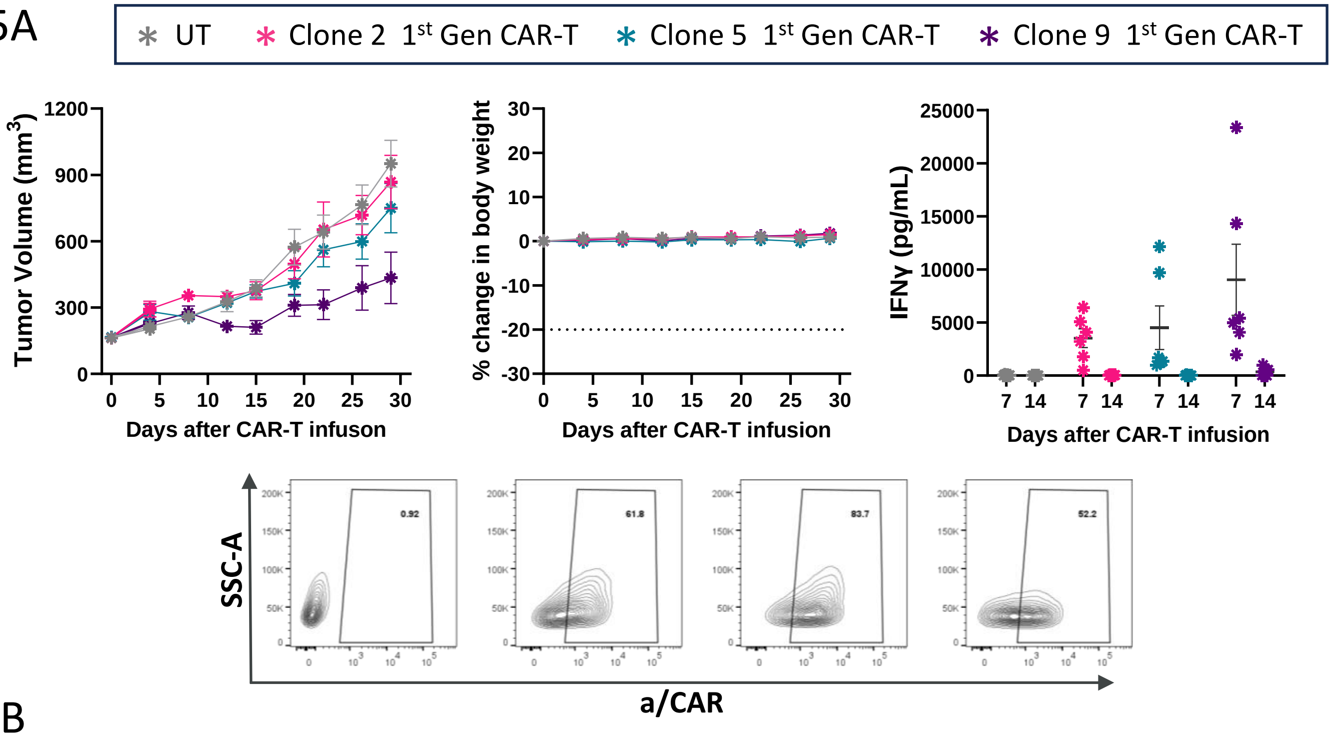

S5B

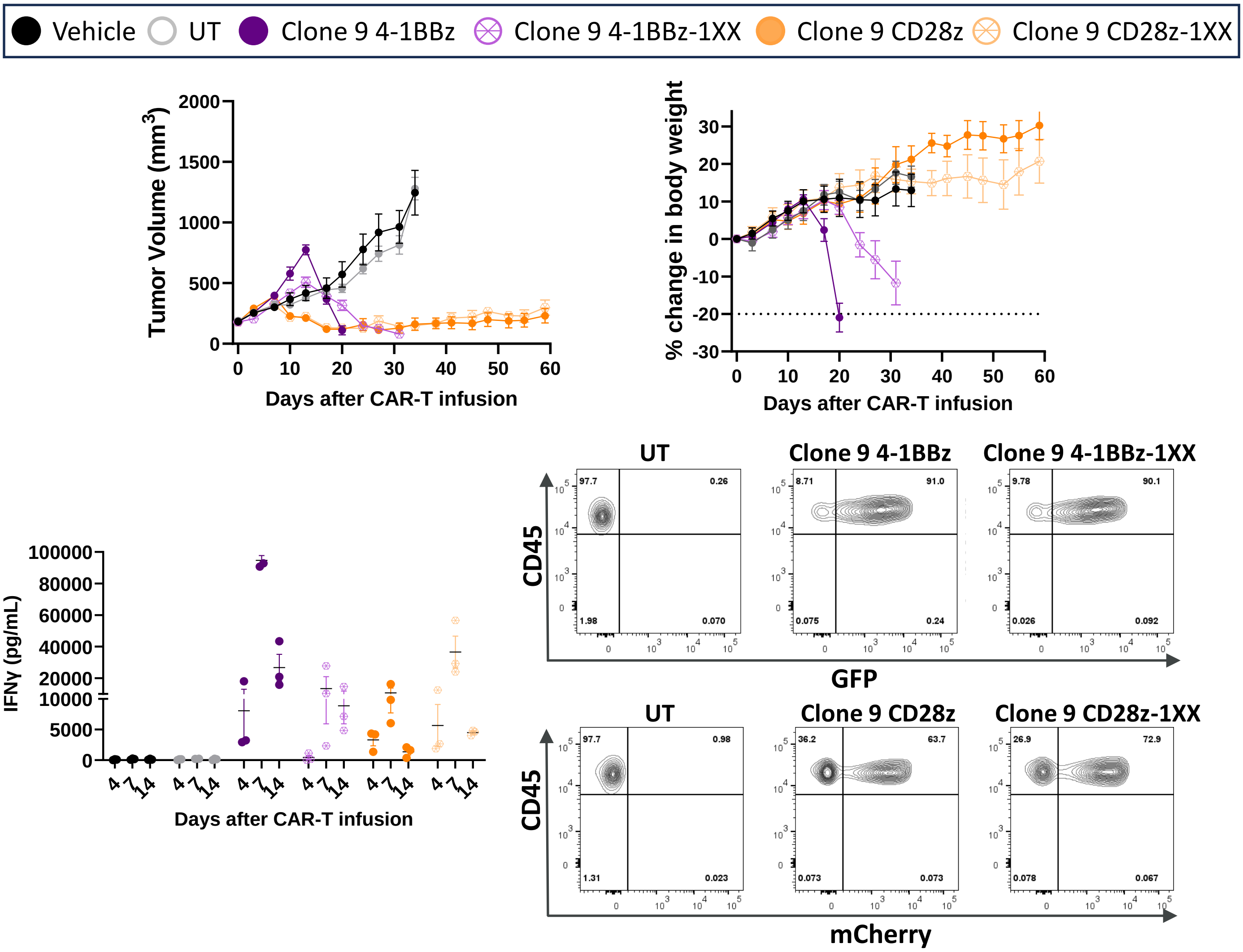

Supplemental Fig. 5

S5A: NSG mice bearing pancreatic PaTu8988s HS xenografts (CLDN18.2 H score = 268) were dosed by tail vein 3e6 first-generation (CD3z only) clone 2, 5 and 9 CAR+ cells or donor matched UT, total T-cell infusion number was matched across groups. Tumor volume and body weight were measured biweekly (n = 6). Serum levels of IFNγ at 7 and 14 days after infusion (n=6). Representative FC plots of CAR surface expression at day 12 post lentiviral transduction compared to UT control. All data represent mean ± SEM of replicates.

S5B: NSG mice bearing PaTu8988s HS xenografts were dosed as described in S5A with 3e6 clone 9 CD28z CD3z WT, CD28z CD3z-1XX, Bz CD3z WT or Bz CD3z-1XX CAR-T, donor matched UT or vehicle (n=5-6). Tumor volume and body weight were measured biweekly (n = 6). Serum levels of IFNγ at 4, 7 and 14 days after infusion (n=3). Representative FC plots of CAR surface expression at day 12 post lentiviral transduction compared to UT control. All data represent mean ± SEM of replicates.

Supplemental Fig. 6

S6A

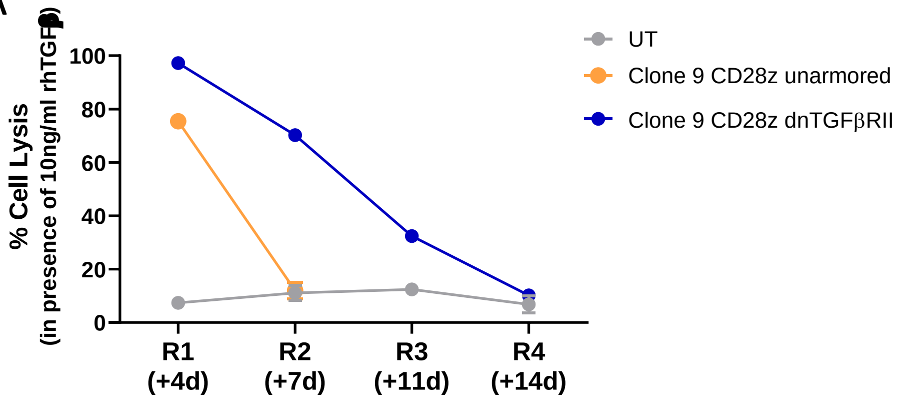

Supplemental Fig. 6

S6A: Serial restimulation assay to examine cytotoxicity and persistence of dnTGFβRII CAR-T and unarmored CAR-T, in presence of 10 ng/ml rhTGFβ. CAR-T were cocultured at 1:2 with BxPC3 + CLDN18.2, tumor lysis was measured every 3–4 days.

Supplemental Fig. 7

PANC_20

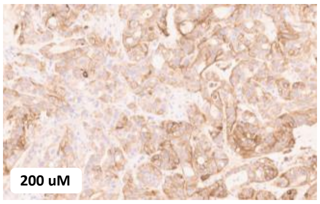

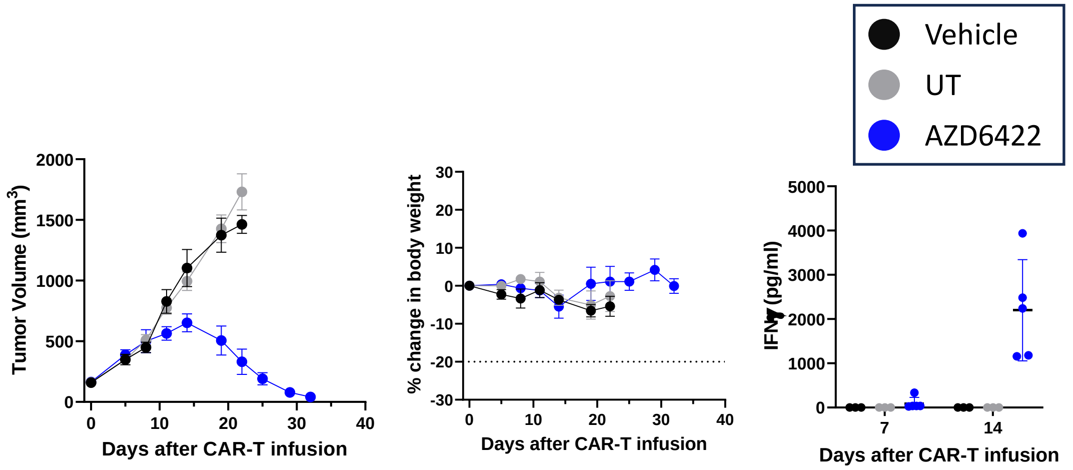

CLDN18.2 H score = 170
TGFβ low

PANC_23

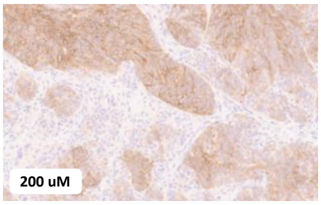

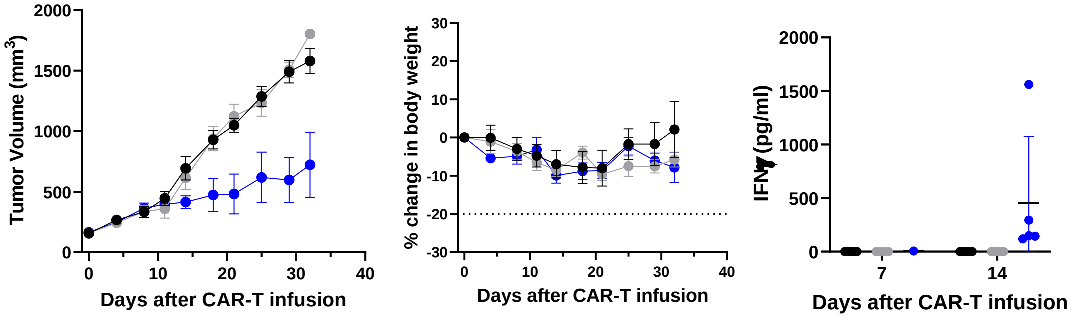

CLDN18.2 H score = 140
TGFβ intermediate

PANC_06

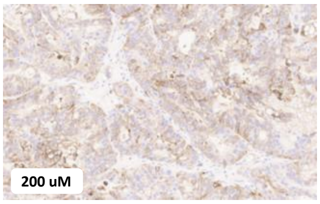

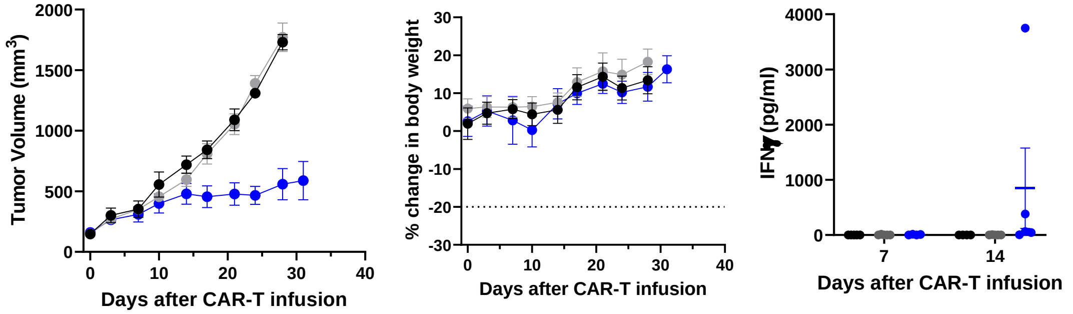

CLDN18.2 H score = 124
TGFβ high

Supplemental Fig. 7

Additional PDX models (PANC_06, PANC_20 and PANC_23) and in vivo antitumor activity of AZD6422 across a range of CLDN18.2 (shown at 10X scan) and TGFβ expression. Mice received a single tail vein infusion of 1e6 AZD6422, donor-matched UT, or vehicle when the average tumor volume reached 150 mm^3. Tumor volumes and body weights were measured biweekly until study completion on day 35, blood was collected for cytokine analysis on days 7 and 14. Data are shown as mean ± SEM (n = 5).
